# Oncogenic Kit signals on endolysosomes and endoplasmic reticulum are essential for neoplastic mast cell proliferation

**DOI:** 10.1038/ncomms6715

**Published:** 2014-12-10

**Authors:** Yuuki Obata, Shota Toyoshima, Ei Wakamatsu, Shunichi Suzuki, Shuhei Ogawa, Hiroyasu Esumi, Ryo Abe

**Affiliations:** 1 Division of Immunobiology, Research Institute for Biomedical Sciences, Tokyo University of Science, Noda, Chiba 278-0022, Japan; 2 Division of Clinical Research, Research Institute for Biomedical Sciences, Tokyo University of Science, Noda, Chiba 278-0022, Japan; 3 National Cancer Center Hospital East, Kashiwa, Chiba 277-8577, Japan

## Abstract

Kit is a receptor-type tyrosine kinase found on the plasma membrane. It can transform mast cells through activating mutations. Here, we show that a mutant Kit from neoplastic mast cells from mice, Kit(D814Y), is permanently active and allows cells to proliferate autonomously. It does so by activating two signalling pathways from different intracellular compartments. Mutant Kit from the cell surface accumulates on endolysosomes through clathrin-mediated endocytosis, which requires Kit’s kinase activity. Kit(D814Y) is constitutively associated with phosphatidylinositol 3-kinase, but the complex activates Akt only on the cytoplasmic surface of endolysosomes. It resists destruction because it is under-ubiquitinated. Kit(D814Y) also appears in the endoplasmic reticulum soon after biosynthesis, and there, can activate STAT5 aberrantly. These mechanisms of oncogenic signalling are also seen in rat and human mast cell leukemia cells. Thus, oncogenic Kit signalling occurs from different intracellular compartments, and the mutation acts by altering Kit trafficking as well as activation.

## Abstract (web summary)

Activating mutations of the tyrosine kinase Kit are commonly found in mast cell neoplasms and gastrointestinal stromal tumours. Here the authors show that mutant Kit, through the activation of PI3K and STAT3 pathways, elicits proliferative and survival signals from endolysosomes and from the endoplasmic reticulum.

## Graphical abstract

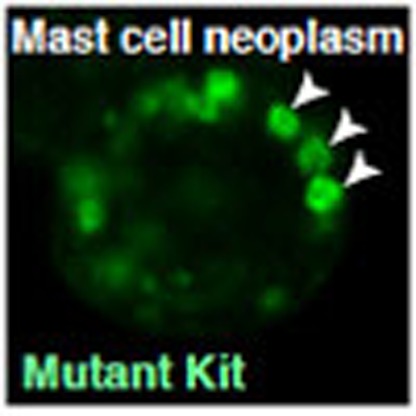

The *Kit* proto-oncogene encodes a type III receptor tyrosine kinase (RTK), a class of proteins that includes platelet-derived growth factor receptors (PDGFR), Fms, and Fms-like tyrosine kinase 3 (Flt3)^1^,^2^,^3^. Kit is expressed on mast cells, interstitial cells of Cajal, haematopoietic cells, germ cells and melanocytes^4^. On stimulation with stem cell factor (SCF), Kit triggers many signalling events at the plasma membrane (PM), resulting in cell proliferation, survival and differentiation^5^.

Kit is composed of five *N*-glycosylated immunoglobulin domains in the N-terminal extracellular portion that bind SCF, as well as a transmembrane domain, and an intracellular C-terminal tyrosine kinase domain^6^. The binding of SCF autophosphorylates Kit on specific tyrosine residues. Kit then binds to other cytoplasmic enzymes containing Src hohomogy-2 (SH2) domain, and this complex phosphorylates other proteins^3^,^4^,^5^,^6^. This process activates the phosphatidylinositol 3-kinase-Akt (PI3K-Akt) pathway, the Ras-Raf-Erk cascade and Src kinases, which regulate cell growth, gene expression and cytoskeletal structures^7^,^8^,^9^,^10^.

In many mast cell neoplasms and gastrointestinal stromal tumours, Kit has gain-of-function mutations, causing permanent, ligand-independent activation of the receptor^11^,^12^,^13^,^14^. In human neoplastic mast cell disorders such as mastocytosis and mast cell leukemia, Kit often has an Asp816Val substitution (D816V) in the kinase domain^12^,^13^. Similar mutations are also found in rat mast cell leukemia (RBL-2H3; D817Y) and a mouse mastocytoma (P815; D814Y)^13^. Their permanent activation of the PI3K-Akt pathway causes neoplastic transformation. When mutant Kit activates the PI3K-Akt pathway, this suppresses apoptotic proteins such as Bim, resulting in cell survival^15^,^16^,^17^,^18^. When mutant Kit phosphorylates signal transduction and activator of transcription (STAT) proteins, they move from the cytoplasm to the nucleus and cause transcription of proto-oncogenes such as *c-myc*^19^,^20^,^21^.

Cancer-causing mutants of RTKs, such as Met(D1246N) and Flt3-internal tandem duplication (Flt3-ITD), can cause incorrect signalling not just from the PM, but also from intracellular compartments, because their activated kinase domain is always exposed^22^,^23^,^24^,^25^,^26^. These mutations also change the trafficking and degradation of the receptors, because they change glycosylation and ubiquitination, and receptors accumulate in the wrong compartments^22^,^23^,^24^,^25^,^26^,^27^. Signalling by oncogenic RTKs on intracellular compartments has been implicated in their transforming ability, but the mechanism of signalling by mutant Kit is not fully understood.

We recently established two mast cell lines, RCM and R, from mouse splenocytes. RCM cells proliferate without cytokines, generate tumours *in vivo* and express a mutant Kit, Kit(D814Y). R cells require cytokines to proliferate and express wild-type Kit (Kit(wt)). This scenario allows us to compare Kit(wt) with Kit(D814Y) in an identical cellular background.

To explore how Kit(D814Y) transduces oncogenic signals, we studied what pathways it activates, from various subcellular compartments, using immunofluorescence confocal microscopy, vesicle immunoprecipitation and chemical inhibition of intracellular trafficking.

In mice cells, Kit(D814Y) from the PM accumulates on endolysosomes through clathrin-mediated endocytosis (CME); this occurs in a kinase activity-dependent manner. It then forms a complex with PI3K, and activates Akt, leading to cell proliferation. Also, soon after Kit(D814Y) is synthesized, it appears in the endoplasmic reticulum (ER), where it causes oncogenic activation of STAT5. Two other mast cell lines, HMC-1 and RBL-2H3, from humans and rats, gave similar results. Our findings demonstrate that Kit signalling from subcellular compartments is necessary for the neoplastic proliferation of mast cells.

## Results

### Kit^D814Y^ causes autonomous proliferation of mouse RCM cells

**Figure 1:**
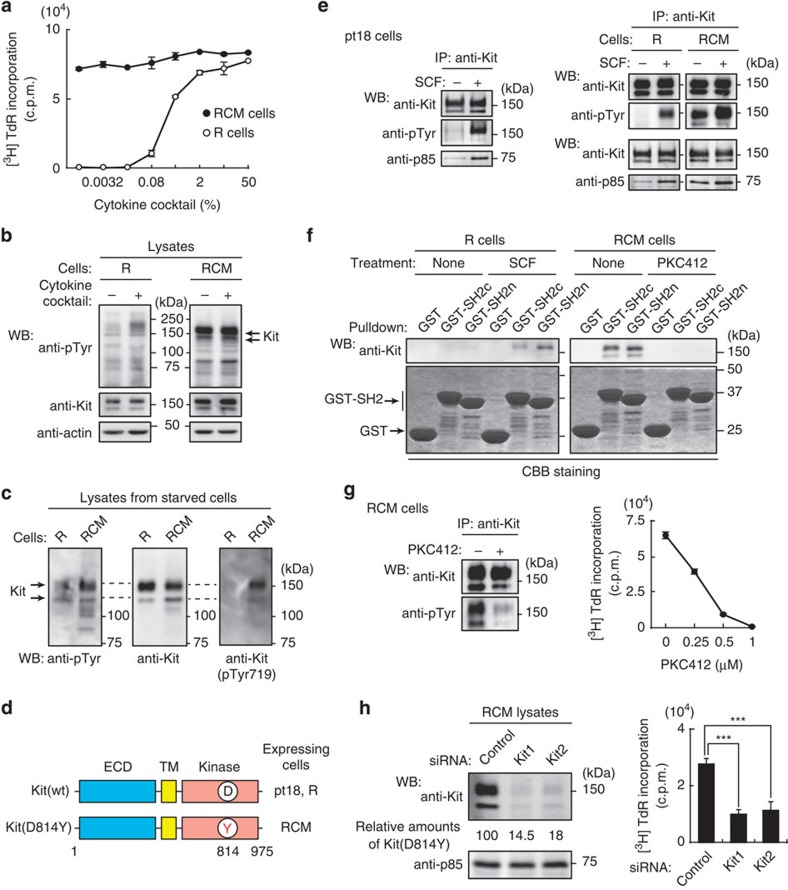
Kit(D814Y) is essential for autonomous proliferation of mouse RCM cells. (**a**) RCM and R cells were cultured in the indicated cytokine cocktail for 48 h. Proliferation was assessed by [^3^H]-thymidine incorporation into R (open circles) and RCM cells (filled circles). Results (c.p.m.) are means±s.d. (*n*=3). NB: RCM cells proliferated without the cytokine cocktail. (**b**,**c**) Expression of constitutively phosphorylated Kit in RCM cells. Starved R and RCM cells were treated with the cytokine cocktail for 5 min. Lysates were immunoblotted with anti-phosphotyrosine (anti-pTyr), anti-Kit, anti-Kit(pTyr719) and anti-actin. Arrows indicate constitutively tyrosine-phosphorylated protein corresponding to Kit in RCM cells. (**d**) Schematic representations of normal Kit and Kit(D814Y) showing the extracellular domain (ECD), the transmembrane domain (TM), the kinase domain, Asp814 in the kinase domain (D in black) and the tyrosine mutation at 814 (Y in red). (**e**) Constitutive activation of Kit(D814Y). Starved pt18, R, and RCM cells were treated with 50 ng ml^−1^ SCF for 5 min. Kit was immunoprecipitated then immunoblotted. (**f**) GST-pulldown. RCM or R cells were treated with 1 μM PKC412 (Kit kinase inhibitor) for 24 h or 50 ng ml^−1^ SCF for 5 min, respectively. Kit was pulled down with GST-SH2c or GST-SH2n, and amounts of Kit pulled down was assayed by immunoblotting. GST proteins were visualized by Coomassie staining. (**g**) Effect of PKC412 on the kinase activity of Kit(D814Y). RCM cells treated with 1 μM PKC412 were cultured for 24 h. Anti-Kit immunoprecipitates were immunoblotted. The graph shows the levels of [^3^H]-thymidine incorporation into RCM cells at the indicated PKC412 concentrations. Results (c.p.m.) are means±s.d. (*n*=3). (**h**) RCM cells were transfected with control siRNA or Kit siRNAs (Kit1 or Kit2) and cultured for 20 h. Lysates were immunoblotted with anti-Kit and anti-p85. Amounts of Kit(D814Y) are expressed relative to control cell lysate, after normalization with p85. The graph shows the levels of [^3^H]-thymidine incorporation into RCM cells. Results (c.p.m.) are means±s.d. (*n*=3). Data were subjected to one-way ANOVA with Dunnett’s multiple comparison *post-hoc* test. ***P<0.001.

We recently established two mast cell lines from mouse splenocytes, RCM cells and R cells, bearing c-Kit and FcεRI. RCM cells grow without cytokines and develop tumours *in vivo* (Fig. 1a). These cells show constitutively tyrosine-phosphorylated 145- and 160-kDa proteins, identified as the Kit tyrosine kinase (Fig. 1b,c; see also Fig. 4b). Furthermore, Kit’s kinase domain has an Asp814Tyr (D814Y) mutation (Fig. 1d), which keeps the kinase permanently active^12^,^13^,^21^.

Immunoprecipitation assays confirmed that Kit(wt) in R cells and pt18 cells^28^ was activated in a ligand-dependent manner, whereas Kit(D814Y) was phosphorylated and associated with the PI3K p85 subunit without SCF (Fig. 1e; see also Fig. 3i), and thus was permanently active. Glutathione *S*-transferase-pulldown (GST-pulldown) assays showed that the C-terminal or N-terminal SH2 domains of p85 (SH2c or SH2n) were associated with SCF-stimulated Kit(wt) and Kit(D814Y) (Fig. 1f). Next, we treated RCM cells with PKC412, an inhibitor of Kit tyrosine kinase^29^. Similar to previous reports^16^,^29^, PKC412 inhibited the phosphorylation of Kit(D814Y) and p85’s association with Kit(D814Y), and also inhibited cell proliferation in a dose-dependent manner (Fig. 1f,g and Supplementary Fig. 1; see also Fig. 4c). We next performed RNA interference experiments to suppress Kit(D814Y). Western blotting showed 80–85% less Kit(D814Y) protein after transfection with Kit1 and Kit2 small interfering RNAs (siRNAs) for 20 h (Fig. 1h, left). Knockdown of Kit(D814Y) also suppressed cell proliferation (Fig. 1h, right). Thus, the kinase activity is required for autonomous proliferation.

### Kit^D814Y^ localizes to endolysosomes

**Figure 2:**
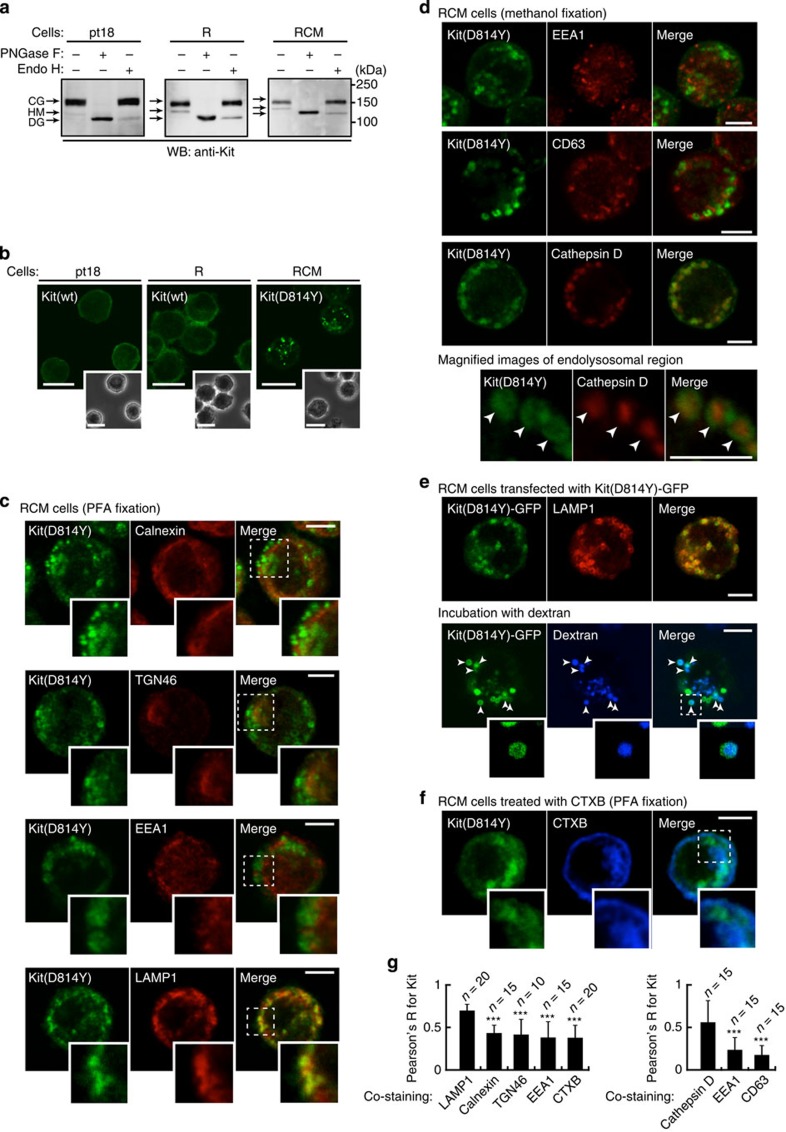
Kit(D814Y) localizes to endolysosomes in mouse RCM cells. (**a**) Glycosylation of Kit(D814Y). Lysates from pt18, R and RCM cells were treated with peptide N-glycosidase F (PNGase F) or endoglycosidase H (endo H), then immunoblotted with anti-Kit. CG, complex-glycosylated form; DG, deglycosylated form; HM, high mannose form. NB: most Kit(D814Y) was a mature complex-glycosylated form. (**b**) Localization of normal Kit and Kit(D814Y). RCM, R or pt18 cells were fixed with PFA and stained with anti-Kit. Phase contrast images are shown. Bars, 10 μm. NB: normal Kit accumulated at the PM; Kit(D814Y) at vesicular structures. (**c**–**g**) Localization of Kit(D814Y) to endolysosomes. (**c**) PFA-fixed or (**d**) methanol-fixed RCM cells were double-stained with anti-Kit (green) in conjunction with the indicated antibody (red). Insets show the magnified images of the boxed area. Representative images of Kit-positive endolysosomes containing cathepsin D are shown. Bars, 5 μm. CD63, cluster of differentiation 63; EEA1, early endosome antigen-1; LAMP1, lysosome-associated membrane protein-1; TGN46*, trans*-Golgi network 46. (**e**) Endolysosomal localization of Kit(D814Y)-GFP. RCM cells transfected with Kit(D814Y)-GFP. (Upper panels) Cells were stained with anti-LAMP1 (endolysosome marker; red). (Lower panels) For visualizing endocytic compartments, cells were incubated with 1 mg ml^−1^ AF647-dextran for 1 h. Expressed protein and dextran were visualized by GFP (green) and AF647 fluorescence (blue). Magnified images of the boxed area are shown. Arrowheads indicate Kit-positive endolysosomes that contain dextran. Bars, 5 μm. NB: Kit(D814Y) surrounded cathepsin D and dextran. (**f**) For visualizing recycling endosomes, RCM cells were incubated for 1 h with 5 μg ml^−1^ AF647-cholera toxin subunit B (AF647-CTXB) and stained with anti-Kit (green). The toxin was visualized with AF647 fluorescence (blue). Magnified images of the boxed area are shown. Bar, 5 μm. (**g**) Pearson’s coefficients (Pearson’s R) were calculated by intensity correlation analysis of Kit(D814Y) versus organelle markers. Results are means±s.d. (*n*=10~20). Data were subjected to one-way ANOVA with Dunnett’s multiple comparison *post-hoc* test. ***P<0.001.

Kit mutants exogenously expressed in CHO, NIH3T3 or HEK293 cells are found mainly in the Golgi apparatus or ER in an immaturely glycosylated form^30^,^31^,^32^. To investigate Kit’s glycosylation state, we treated Kit(D814Y) from RCM cells with endoglycosidase H, which digests immature high-mannose forms, but not mature complex-glycosylated forms. Figure 2a shows that most Kit(D814Y), like Kit(wt), was present in a complex-glycosylated form. Kit(wt) and Kit(D814Y) both shifted to a non-glycosylated form following the complete digestion of N-linked glycans by peptide-N-glycosidase F.

Next, we investigated the subcellular localization of Kit in paraformaldehyde-fixed (PFA-fixed) cells by immunofluorescence confocal microscopy. Consistent with previous reports^9^,^33^,^34^, in pt18 and R cells, most Kit(wt) was at the PM, whereas in RCM cells Kit(D814Y) was mainly on vesicular structures (Fig. 2b). We investigated these structures and calculated Pearson’s correlation coefficients (Pearson’s R) between Kit and various markers: calnexin (ER), TGN46 (Golgi), EEA1, CD63 (endosome), LAMP1 and cathepsin D (endolysosome). As cathepsin D and CD63 could not be detected by immunofluorescence in PFA-fixed cells, we fixed RCM cells with methanol to visualize those proteins. Although Kit’s distributions were largely similar between the different methods (Supplementary Fig. 2a,b), methanol caused fluorescence loss in the cytosol and nucleus (compare Fig. 2c with 2d). Kit(D814Y) co-localized with LAMP1 significantly, and with cathepsin D-positive vesicles somewhat, suggesting it is mainly present on endolysosomes (Fig. 2c,d,g). Moreover, green fluorescent protein-tagged (GFP-tagged) Kit(D814Y) was co-localized with LAMP1, and with fluorescent dextran, which is incorporated into endocytic compartments^35^ (Fig. 2e). Kit(D814Y) surrounded cathepsin D as well as dextran (Fig. 2d,e, magnified images), indicating that the kinase domain is exposed on the cytoplasmic surface of endolysosomes.

Next, we compared the distribution of Kit(D814Y) and recycling endosomes using cholera toxin subunit B (CTXB; a recycling endosome marker)^36^. Kit’s localization to recycling endosomes was significantly less than that to endolysosomes (Fig. 2f,g), and a second marker of recycling endosomes, Rab11, confirmed this (Supplementary Fig. 2c). These results indicate that Kit(D814Y) traffics to endolysosomes rather than recycling to the PM.

### Kit^D814Y^ moves to endolysosomes by kinase–mediated endocytosis

**Figure 3:**
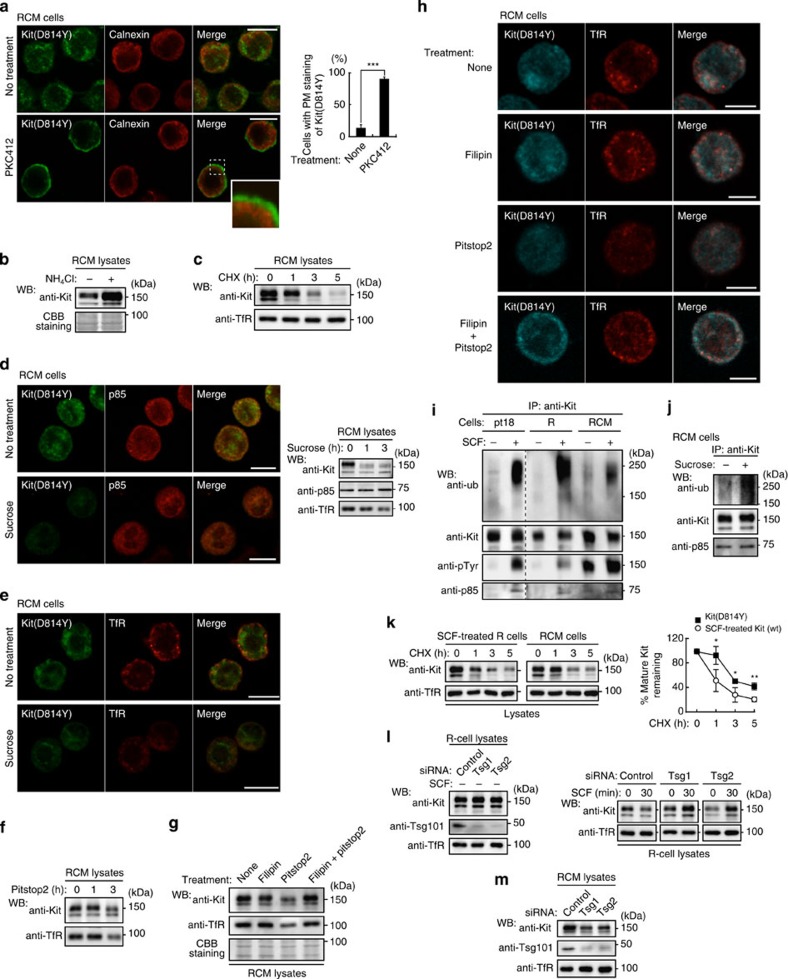
Kit(D814Y) traffics from the plasma membrane to endolysosomes through its kinase activity and clathrin-mediated endocytosis. (**a**) RCM cells cultured in the presence of 1 μM PKC412 (Kit inhibitor) for 24 h and stained with anti-Kit (green) and anti-calnexin (ER marker; red). Bars, 10 μm. The graph shows the percentage of cells with predominant PM localization of Kit(D814Y). Results (%) represent means±s.d. from three independent experiments (*n*>200 cells). ***P<0.001, Student’s *t*-test. (**b**,**c**) RCM cells were cultured in the presence of (**b**) 20 mM NH_4_Cl (inhibits lysosomal proteases) for 24 h or (**c**) 200 μg ml^−1^ cycloheximide (inhibits protein synthesis) for the indicated periods, then immunoblotted. Total protein levels were confirmed by Coomassie staining. (**d**–**f**) Inhibition of clathrin-mediated endocytosis by sucrose or pitstop2. RCM cells were treated with (**d**,**e**) 0.45 M sucrose or (**f**) 50 μM pitstop2. After 3 h, cells were stained with antibody. Bars, 10 μm. (**f**) Immunoblots. (**g**,**h**) Inhibition of non-clathrin endocytosis by filipin. RCM cells treated with 1 μg ml^−1^ filipin and/or 50 μM pitstop2 were cultured for 3 h. (**g**) Lysates were immunoblotted. Total protein levels were confirmed by Coomassie staining. (**h**) Cells were stained with anti-Kit (cyan) and anti-TfR (red). Bars, 5 μm. (**i**,**j**) Ubiquitination of Kit(D814Y). Starved pt18, R and RCM cells were treated with 50 ng ml^−1^ SCF (**i**) or 0.45 M sucrose (**j**) for 5 min. Anti-Kit immunoprecipitates were immunoblotted. Ub: ubiquitin. (**k**) RCM or SCF-treated R cells were cultured in 200 μg ml^−1^ cycloheximide (CHX) for the indicated periods, then immunoblotted. The graph shows the percentage of mature Kit remaining after CHX treatment. Results (%) represent means±s.d. from three independent experiments. *P<0.05; **P<0.01, Student’s *t*-test. (**l**,**m**) R cells or RCM cells were transfected with Tsg101 siRNAs (Tsg1 or Tsg2) and cultured for 24 h. Lystes from R cells treated with 50 ng ml^−1^ SCF for 30 min (**l**) or RCM cells (**m**) were immunoblotted.

In RCM cells treated for 24 h with the kinase inhibitor PKC412, there was more Kit(D814Y) at the PM and less in endolysosomes (Fig. 3a). Treatment did not cause accumulation of Kit(D814Y) on either ER or Golgi. Following removal of inhibitor, Kit(D814Y) moved within 60 min from the PM, via endosomes, to endolysosomes (Supplementary Fig. 3a). These results suggest that Kit’s kinase activity is needed for endocytosis, but not for biosynthetic traffic to the PM. Figure 3b shows that 24-h treatment with an inhibitor of lysosomal proteases (NH_4_Cl) resulted in retention of Kit(D814Y) inside RCM cells, indicating that endocytosed Kit(D814Y) is targeted to lysosomes, and subsequently degraded. In the presence of cycloheximide, an inhibitor of protein synthesis, Kit(D814Y) was degraded within 5 h, whereas transferrin receptor (TfR), which undergoes recycling, was unchanged (Fig. 3c), suggesting that Kit traffics constitutively to lysosomes.

In endocytosis of growth factor receptors, for example, of epidermal growth factor (EGF) and transforming growth factor β (TGFβ), clathrin plays a critical role in their stability^37^,^38^. Accordingly, receptor-mediated endocytosis is divided into two major categories: non-clathrin endocytosis (NCE) and CME^37^,^38^,^39^. To study the role of clathrin in Kit(D814Y) endocytosis, we treated RCM cells with hypertonic sucrose, thereby blocking the formation of clathrin-coated pits^40^. Within 3 h, this reduced the protein levels of mature Kit(D814Y) and TfR without affecting the cytosolic protein p85 (Fig. 3d,e). Similar results were obtained from RCM cells treated with pitstop2, a selective inhibitor of CME^41^ (Fig. 3f). To confirm that, with CME blocked, Kit and TfR were being processed by NCE, we treated cells for 3 h with pitstop2 plus filipin, or with sucrose plus filipin, which blocks NCE by disrupting lipid rafts^37^,^42^. Kit(D814Y) and TfR were then found at the PM, presumably protected from rapid degradation (Fig. 3g,h and Supplementary Fig. 3b). In cells treated with filipin alone, the protein levels and localization of Kit(D814Y) were unaffected (Fig. 3g,h and Supplementary Fig. 3b,c). Similar to sucrose and pitstop2, knockdown of the assembly polypeptide-2 α subunit (AP2α), which is required for CME^39^, decreased the protein levels of Kit(D814Y) and TfR (Supplementary Fig. 3d). Thus, the major pathway for Kit(D814Y) endocytosis is CME.

In NCE, ubiquitination of receptors and other endocytic cargo is important for association with the ESCRT (endosomal sorting complexes required for transport) machinery, for rapid incorporation into lysosomes and for degradation^39^,^43^,^44^,^45^. As Kit(D814Y) mainly undergoes CME not NCE, we hypothesized that it may not be fully ubiquitinated. When Kit(wt) was stimulated with SCF, in pt18 and R cells it became fully ubiquitinated, and was degraded within 30 min (Figs 3i and 6g; see also Fig. 3l), consistent with previous reports^31^,^32^,^33^,^34^,^45^. In sharp contrast, in RCM cells ubiquitination of Kit(D814Y) was much lower, regardless of the presence or absence of SCF. When CME was blocked, Kit(D814Y) became substantially ubiquitinated (Fig. 3j), presumably as it then trafficked via NCE. These results suggest that, in RCM cells, most Kit(D814Y) undergoes CME depending on its kinase activity, and accumulates on the cytoplasmic surface of endolysosomes, but not in a fully ubiquitinated state. In support of this, degradation of Kit(D814Y) was significantly slower than that of activated Kit(wt) in the presence of cycloheximide (Fig. 3k).

Next, to investigate the mechanism of endosome-to-lysosome trafficking of Kit, we knocked down tumour-susceptibility gene 101 (Tsg101), a component of ESCRT^43^,^44^,^46^. Figure 3l shows that in R cells treated with SCF for 30 min, Tsg101 knockdown inhibited the degradation of Kit(wt) but not of TfR (see also Supplementary Fig. 5e). These results suggest that Kit(wt) is incorporated into lysosomes in a manner that is dependent on ESCRT, similar to EGFR^39^,^43^,^46^. By contrast, knockdown decreased Kit(D814Y) in RCM cells (Fig. 3m). As ESCRT depletion enhances ESCRT-independent transport into lysosomes^46^, our results indicate that Kit(D814Y) is incorporated into lysosomes in a manner that is independent of ESCRT.

### Activation of Akt and STAT5 is necessary for proliferation of RCM cells

Neoplastic transformation of mast cells involves phosphorylation and activation of Akt, STAT proteins, Erk1/2 and JAKs^4^,^13^,^15^,^16^,^17^,^18^,^19^,^20^,^21^. In RCM cells, Akt and STAT5, but not Erk1/2, STAT3 and JAK2, were constitutively phosphorylated (Supplementary Fig. 4a,b).

**Figure 4:**
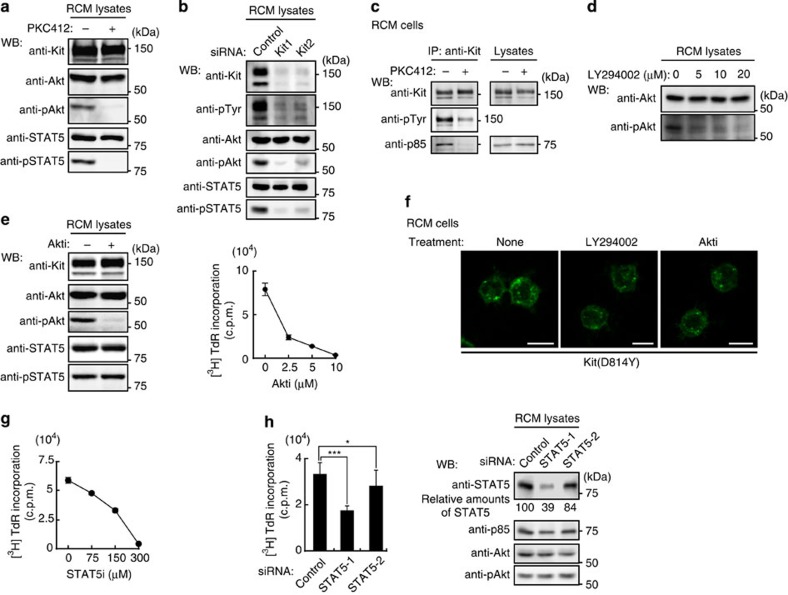
In mouse RCM cells, Akt and STAT5 must be permanently active for autonomous proliferation. (**a**,**b**) Constitutive activation of Akt and STAT5 by Kit(D814Y). (**a**) RCM cells were treated with 1 μM PKC412 (Kit kinase inhibitor) for 24 h, lysed, then immunoblotted. (**b**) RCM cells transfected with control siRNA or Kit siRNAs were cultured for 20 h, then immunoblotted. (**c**) Association of Kit(D814Y) with PI3K. RCM cells were treated with 1 μM PKC412 for 24 h. Anti-Kit immunoprecipitates (left) and lysates (right) were then immunoblotted. NB: Kit(D814Y) was dissociated from p85 by PKC412 treatment. (**d**) Activation of Akt by PI3K. RCM cells were treated with the PI3K inhibitor LY294002. Lysates were then immunoblotted. (**e**) Role of Akt in proliferation of RCM cells. RCM cells treated with 10 μM Akti (Akt inhibitor) were cultured for 24 h. (Left) Lysates were then immunoblotted. (Right) [^3^H]-thymidine incorporation. Results (c.p.m.) represent means±s.d. (*n*=3). (**f**) Effect of LY294002 and Akti on Kit(D814Y) localization. RCM cells were treated with 20 μM LY294002 or 10 μM Akti for 24 h and stained with anti-Kit. Bars, 10 μm. (**g**,**h**) Role of STAT5 in proliferation of RCM cells. (**g**) [^3^H]-thymidine incorporation into RCM cells treated with the STAT5 inhibitor STAT5i for 24 h. Results (c.p.m.) are means±s.d. (*n*=3). (**h**) RCM cells transfected with control siRNA or STAT5 siRNAs (STAT5-1 and STAT5-2) were cultured for 24 h. (Left) Bar graph shows the levels of [^3^H]-thymidine incorporation into RCM cells. Results (c.p.m.) are means±s.d. (*n*=11). Data were subjected to one-way ANOVA with Dunnett’s multiple comparison *post-hoc* test. *P<0.05; ***P<0.001. (Right) Lysates were immunoblotted. Amounts of STAT5 are expressed relative to control cell lysate after normalization with p85.

To investigate whether it was Kit(D814Y) that had activated Akt and STAT5, we inhibited Kit(D814Y) by PKC412 or siRNA-mediated knockdown. Both treatments inhibited the activation of Akt and STAT5 (Fig. 4a,b). We further found that the PI3K p85 subunit was co-immunoprecipitated with Kit(D814Y), and that PKC412 blocked this association (Fig. 4c; see also Fig. 1f, right). In this cell line, a PI3K inhibitor LY294002 suppressed Akt activation in a dose-dependent manner (Fig. 4d), as reported previously^7^,^19^,^47^. Thus, Kit(D814Y) activates Akt through association with PI3K.

Next, we tested whether Akt activation was required for autonomous proliferation. In RCM cells, the inhibitor Akti repressed the activation of Akt without affecting STAT5. It also caused a dose-dependent suppression of cell proliferation (Fig. 4e). Although the PI3K-Akt pathway has a role in RTK trafficking^35^,^48^,^49^, no redistribution was seen here on treatment with either LY294002 or Akti (Fig. 4f). This shows that the PI3K-Akt pathway has an essential role in cell proliferation but does not influence Kit(D814Y) trafficking.

We next suppressed STAT5 function by utilizing STAT5 inhibitor (STAT5i) or knockdown. Both treatments inhibited the proliferation of RCM cells (Fig. 4g,h). Knockdown had no effect on the activation of Akt. On the contrary, proliferation was unaffected by Erk1/2 inhibition with U0126 (Supplementary Fig. 4c). Taken together, these results suggest that Kit(D814Y) constitutively activates the PI3K-Akt pathway and STAT5, and that these activations are essential for autonomous proliferation.

### Kit^D814Y^ activates Akt through PI3K only on endolysosomes

**Figure 5:**
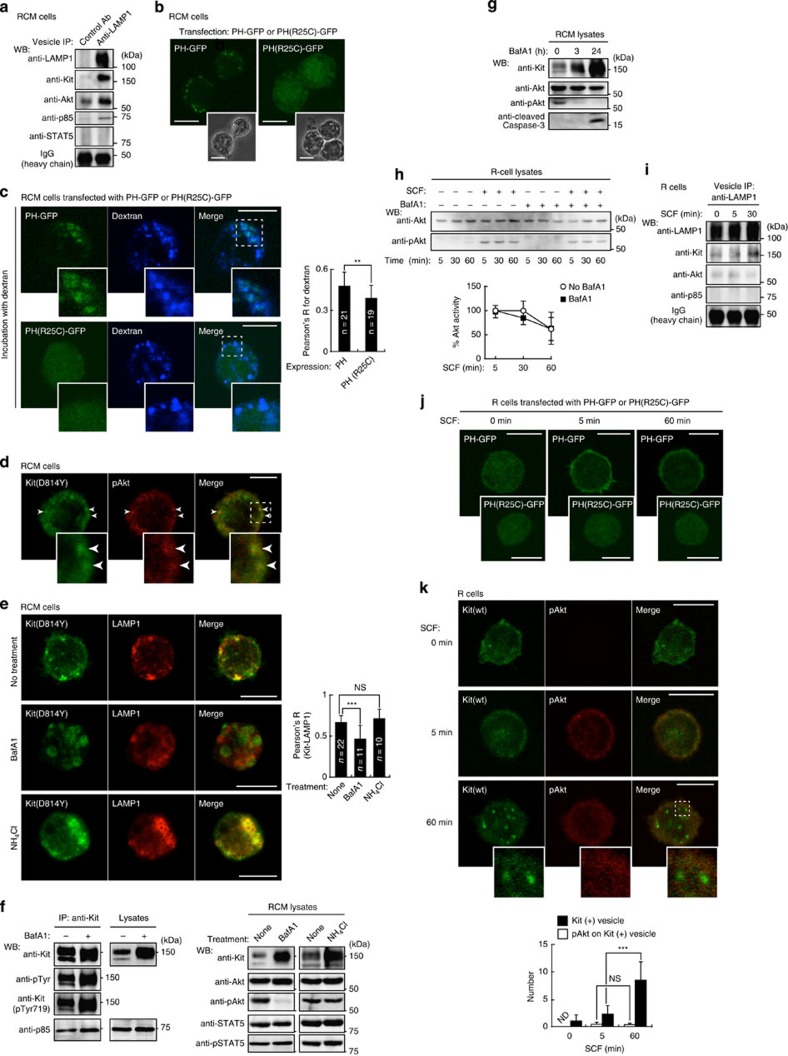
Kit(D814Y) traffics to endolysosomes to activate Akt in mouse cells. (**a**) Endolysosomes from RCM cells were immunoprecipitated with control or anti-LAMP1, then immunoblotted. (**b**,**c**) RCM cells were transfected with PH-GFP or PH(R25C)-GFP. (**b**) Expressed proteins were visualized by GFP fluorescence. Phase contrast images are shown. Bars, 10 μm. (**c**) Cells were cultured for 1 h with 1 mg ml^−1^ AF647-dextran (a marker for endocytic compartments). Bars, 10 μm. The graph shows Pearson’s R correlation coefficient between dextran and expressed proteins. Results are means±s.d. from 19 or 21 cells. **P<0.01, Student’s *t*-test. (**d**) RCM cells were co-stained for Kit(D814Y) (green) and phosphorylated Akt (pAkt; red). Bar, 5 μm. (**e**–**g**) RCM cells were cultured with 100 nM BafA1 (blocks endosomal trafficking) or 20 mM NH_4_Cl (blocks proteases) for 24 h. (**e**) Cells stained with anti-Kit (green) and anti-LAMP1 (endolysosome marker; red). Bars, 10 μm. The graph shows Pearson’s R correlation coefficient between Kit(D814Y) and LAMP1. Results are means±s.d. (*n*=10~22). Data were subjected to one-way ANOVA with Dunnett’s multiple comparison *post-hoc* test. ***P<0.001; NS, not significant. (**f**) Immunoblots of anti-Kit immunoprecipitates and cell lysates. (**g**) Immunoblots, RCM cells treated with 100 nM BafA1 for the indicated periods. (**h**) After 3-h treatment with 100 nM BafA1, R cells were stimulated with 50 ng ml^−1^ SCF for the indicated periods in the presence of BafA1, then immunoblotted. The graph shows the percentage of pAkt after nomalization with control at 5-min stimulation. Results (%) represent means±s.d. from three independent experiments. BafA1 did not affect pAkt significantly (Student’s *t*-test). (**i**) Immunoblots, endolysosomes from R cells treated with 50 ng ml^−1^ SCF as indicated. (**j**,**k**) R cells were stimulated with 50 ng ml^−1^ SCF for 5 or 60 min. Expressed proteins, Kit(wt) or pAkt are shown. Bars, 10 μm. The graph shows the number of Kit(+) vesicles with pAkt. Results (%) represent means±s.d (*n*=8~14). NS, not significant; ***P<0.001, Student’s *t*-test. ND=not detected.

Activated RTKs, such as EGFR, Met, and PDGFR can transduce signals not only on the cell’s outer membrane, but also on endocytic compartments^23^,^24^,^49^,^50^,^51^,^52^. To examine whether endolysosomes serve as a platform for oncogenic Kit signalling, we purified endolysosomes from RCM cells by immunoprecipitation, using an anti-LAMP1 antibody. Figure 5a shows that Kit(D814Y), Akt and p85, but not STAT5, were found in the endolysosomal fraction. As active Akt is bound to phosphatidylinositol-3,4,5-triphosphate (PI(3,4,5)P_3_), which is generated by PI3K, through its pleckstrin homology (PH) domain^3^,^53^, it is likely that Akt and Kit(D814Y)-PI3K become associated in endolysosomes. To test this hypothesis, we investigated whether PI(3,4,5)P_3_ was generated on endolysosomes, by expression of a GFP-tagged Akt PH domain (PH-GFP)^54^. In RCM cells most PH-GFP was on vesicles but not at the PM, whereas PH(R25C)-GFP, a non-PI(3,4,5)P_3_-binding mutant, was in the cytoplasm (Fig. 5b). As PH-GFP could not be detected after immunofluorescence, we directly visualized endocytic compartments by fluorescent dextran. Figure 5c shows that they were significantly co-localized with PH-GFP. Furthermore, phosphorylated Akt was found on vesicles where Kit localized (Fig. 5d). These results suggest that Akt is activated by Kit(D814Y) predominantly on endolysosomes.

Next, we investigated whether Kit(D814Y) must localize to endolysosomes to activate Akt. We used bafilomycin A1 (BafA1), which blocks endosomal trafficking without affecting internalization and recycling^44^,^55^. After 24 h, Kit(D814Y) was rare in endolysosomes (LAMP1-positive), but common in endosomes (EEA1-positive) (Fig. 5e and Supplementary Fig. 5a), indicating that trafficking from endosomes to endolysosomes is blocked. Figure 5f shows that BafA1 treatment also prevented Kit(D814Y) degradation. BafA1 did not affect Kit(D814Y)’s kinase activity (Fig. 5f, left). It reduced activation of Akt but not of STAT5 (Fig. 5f). BafA1 did not suppress the association of Kit(D814Y) with p85 and did not affect Kit phosphorylation at Tyr719 (Fig. 5f, left and Supplementary Fig. 5b), a binding site for p85 (refs 7, 8, 53). Therefore, Kit(D814Y) presumably associates with PI3K before reaching endolysosomes. Treatment with NH_4_Cl, (inhibits lysosomal proteases) for 24 h preserved Kit(D814Y) from degradation, but did not affect its localization to endolysosomes (Fig. 5e, bottom). In contrast to BafA1, NH_4_Cl had no effect on the activation of Akt (Fig. 5f, right), suggesting that Kit must localize to endolysosomes to activate Akt. In support of this conclusion, when Kit(D814Y) trafficking to endolysosomes was blocked by BafA1, p85 and Akt did not co-fractionate with the endolysosomal membrane (Supplementary Fig. 5c).

To examine whether the inhibition of Akt by BafA1 resulted from apoptosis, we performed immunoblotting for cleaved caspase-3, an apoptosis marker^20^. Figure 5g shows that BafA1 treatment for 24 h induced cleavage of caspase-3, but treatment for 3 h did not. The treatment for 3 h also inhibited Akt (Fig. 5g), indicating that the effect of BafA1 on Akt activity resulted from inhibition of Kit trafficking, but not from apoptosis.

Kit(wt) is also transported through the endocytic pathway after SCF binding^33^,^34^. To examine whether Kit(wt) also activated a PI3K-Akt pathway on endolysosomes, we treated R cells with BafA1 for 3 h and then stimulated them with SCF. Akt remained active up to 60 min after SCF stimulation, regardless of the presence or absence of BafA1 (Fig. 5h). Similar results were obtained from pt18 cells (Supplementary Fig. 5d). Figure 5i shows that in R cells stimulated with SCF for 30 min, p85 and Akt were absent from endolysosomes. Furthermore, in SCF-stimulated R cells, phosphorylated Akt and PH-GFP were not detected in endolysosomal vesicles (Fig. 5j,k). Also, accumulation of Kit(wt) on endosomes following Tsg101 knockdown did not activate Akt (Supplementary Fig. 5e), suggesting that Kit(wt) activates the PI3K-Akt pathway transiently, presumably when bound to the PM. However, we could not exclude the possibility that Kit(wt) activates Akt soon after endocytosis.

### Partially glycosylated Kit^D814Y^ on the ER activates STAT5

**Figure 6:**
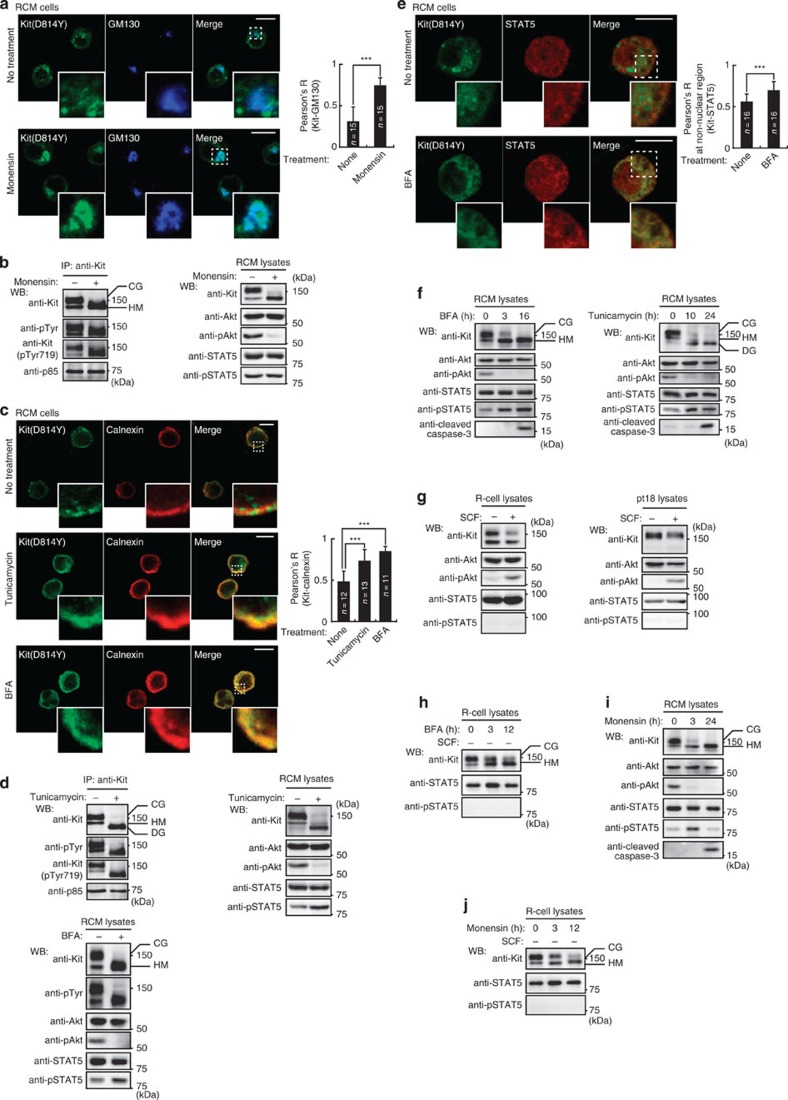
Kit(D814Y) at the ER activates STAT5 in mouse cells. (**a**,**b**) Inhibition of export of Kit(D814Y) from the Golgi. RCM cells were cultured with 250 nM monensin (inhibits Golgi transport) for 24 h. (**a**) RCM cells were stained with anti-Kit (green) and anti-GM130 (Golgi marker, blue). Magnified images of the boxed area are shown. Bars, 10 μm. The graph shows correlation coefficient (Pearson’s R) between Kit and GM130. Results are means±s.d. from 15 cells. ***P<0.001, Student’s *t*-test. (**b**) Immunoblots of anti-Kit immuoprecipitates (left) and cell lysates (right). (**c**–**e**) Inhibition of export of Kit(D814Y) from the ER. RCM cells were cultured with 1 μg ml^−1^ tunicamycin for 24 h, or 5 μM BFA for 16 h to block ER export. (**c**) RCM cells were stained with anti-Kit (green) and anti-calnexin (ER marker; red). Magnified images of the boxed area are shown. Bars, 10 μm. The graph shows the correlation coefficient (Pearson’s R) between Kit and calnexin. Results are means±s.d. from 11 to 13 cells. Data were subjected to one-way ANOVA with Dunnett’s multiple comparison *post-hoc* test. ***P<0.001. (**d**) Immunoblots of anti-Kit immuoprecipitates and cell lysates. NB: ER-localized Kit(D814Y) activated STAT5. (**e**) BFA-treated cells were stained with anti-Kit (green) and anti-STAT5 (red) antibodies. Magnified images of the boxed area are shown. Bars, 10 μm. The graph shows the correlation coefficient (Pearson’s R) between Kit and STAT5 in the non-nuclear region. Results are means±s.d. from 16 cells. ***P<0.001, Student’s *t*-test. NB: accumulation of Kit(D814Y) in the ER significantly enhanced co-localization with STAT5. (**f**) RCM cells were treated with 5 μM BFA, 1 μg ml^−1^ tunicamycin, 250 nM monensin or 100 nM BafA1 for the indicated periods and then immunoblotted. (**g**) Starved R cells (left) or pt18 cells (right) stimulated with 50 ng ml^−1^ SCF for 5 min then lysed and immunoblotted. NB: normal Kit was unable to activate STAT5. (**h**–**j**) RCM cells or starved R cells treated with 250 nM monensin or 5 μM BFA for the indicated periods, then immunoblotted. CG=complex-glycosylated form; DG=deglycosylated form; HM=high mannose form.

Cytochalasin D, an inhibitor of endocytosis through actin depolymerization^35^,^36^, prevented the activation of Akt without influencing Kit(D814Y)’s kinase activity and p85’s association with Kit(D814Y), but the treatment did not affect STAT5 activity (Supplementary Fig. 5b,f). These results suggest that Kit(D814Y) activates STAT5 from a different subcellular compartment. Thus, we determined where in the cell Kit(D814Y) activates STAT5. First, we examined whether exocytic transport of Kit(D814Y) from the Golgi apparatus towards the PM was required. We treated RCM cells with monensin, which inhibits export from the Golgi by blocking intra-Golgi transport^56^. After 24-h treatment, Kit(D814Y) co-localized with the Golgi marker GM130 (Fig. 6a). Figure 6b shows that monensin treatment resulted in expression of partially glycosylated Kit(D814Y). Monensin had no effect on the autophosphorylation of Kit(D814Y), or on p85’s association with Kit(D814Y) (Fig. 6b, left and Supplementary Fig. 5b). Moreover, monensin abolished Akt activation, presumably because Kit could no longer locate to endolysosomes. STAT5 activation was not affected (Fig. 6b, right), suggesting that Kit activates STAT5 on the Golgi and/or ER, not at the PM.

Next, we used tunicamycin to inhibit ER export of Kit(D814Y) by blocking protein glycosylation^26^,^57^. Tunicamycin suppressed trafficking from ER to Golgi, and Kit(D814Y) accumulated on the ER (Fig. 6c, middle panels). Tunicamycin-treated cells also expressed non-glycosylated Kit(D814Y) (Fig. 6d, top). Like monensin, tunicamycin did not stop the autophosphorylation of Kit(D814Y) or p85’s association with Kit(D814Y), but did prevent Akt activation (Fig. 6d, top and Supplementary Fig. 5b). STAT5 activation was again enhanced (Fig. 6d, right), indicating that Kit(D814Y) activates STAT5 on the ER.

Brefeldin A (BFA) also inhibits protein export from the ER^26^,^30^,^58^. On 16-h BFA treatment, as with tunicamycin, partially glycosylated Kit(D814Y) accumulated on the ER and STAT5 became active (Fig. 6c,d). STAT5 was seen in the nucleus and on cytosolic reticular structures, but not at the PM (Fig. 6e, top). BFA treatment significantly enhanced the co-localization of Kit(D814Y) with STAT5 on the reticular structures (Fig. 6e, bottom), further suggesting that Kit(D814Y) activates STAT5 on the ER. Figure 6f shows that the effects of BFA and tunicamycin on Akt and STAT5 resulted from accumulation of Kit(D814Y) at the ER, not from apoptosis. In contrast to Kit(D814Y), in R and pt18 cells, SCF stimulation of Kit(wt) did not activate STAT5 (Fig. 6g), as previously described^27^,^29^. Accumulation of Kit(wt) on the ER did not affect STAT5 activation (Fig. 6h and Supplementary Fig. 5g), indicating that STAT5 activation requires ER-localized Kit(D814Y). Taken together, these results suggest that newly synthesized partially glycosylated Kit(D814Y) on the ER activates STAT5 aberrantly.

Treatment with monensin or BafA1 for 3 h inhibited Akt independently of apoptosis, and activated STAT5 transiently (Figs 5g and 6i; Supplementary Fig. 5h). These drugs did not enhance STAT5 activity in R cells (Fig. 6j and Supplementary Fig. 5i). These results indicate that accumulated Kit(D814Y) on the Golgi and endosomes can activate STAT5 activation transiently. Thus, mechanisms of negative regulation for STAT5 may exist on the Golgi and endosomes.

### The oncogenic role of Kit and its trafficking in rat and human cells

**Figure 7:**
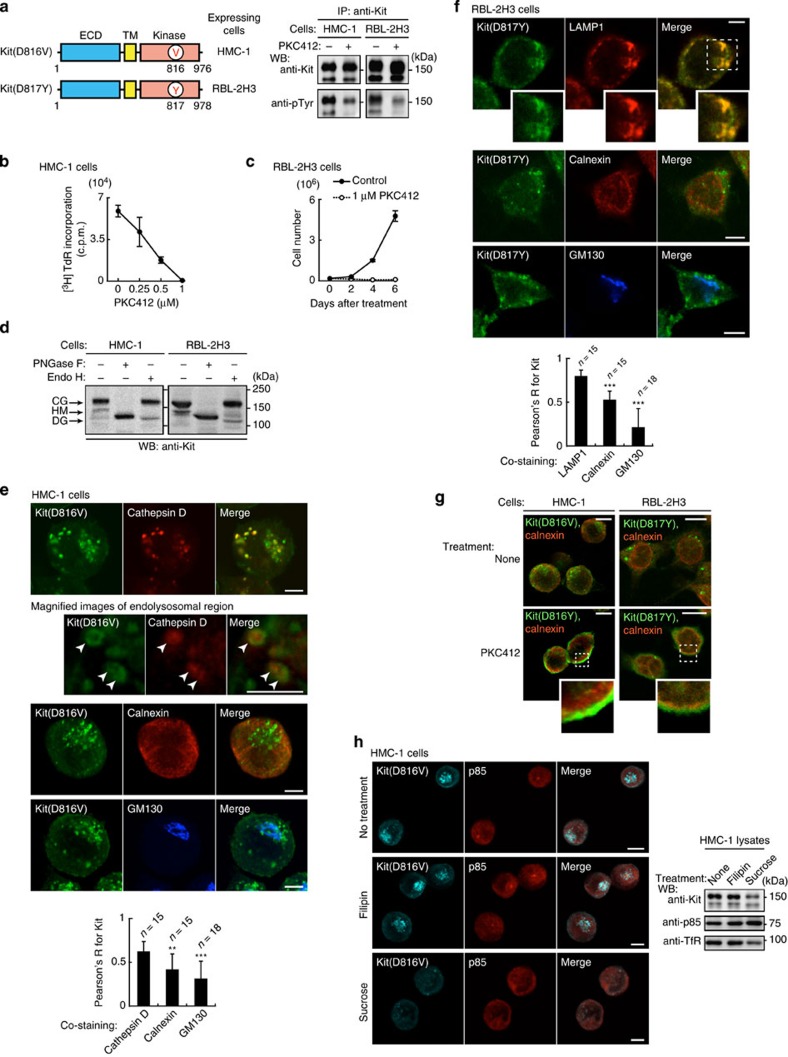
Oncogenic role and intracellular trafficking of mutant Kit in rat and human cells. (**a**) Mutant Kit is constitutively active in HMC-1 and RBL-2H3 cells. (Left) Schematic structures of Kit in HMC-1 and RBL-2H3 cells. (Right) Immunoblots of anti-Kit immunoprecipitates from HMC-1 or RBL-2H3 cells treated with 1 μM PKC412 (Kit kinase inhibitor) for 4 or 12 h, respectively. (**b**,**c**) The effect of PKC412 on proliferation. (**b**) [^3^H]-thymidine incorporation in HMC-1 cells treated with PKC412 for 24 h. Results (c.p.m.) are means±s.d. (*n*=3). (**c**) Growth of RBL-2H3 cells treated with (filled circles) or without (open circles) 1 μM PKC412. Results are means±s.d. (*n*=3). (**d**) Glycosylation of Kit(D816V) and Kit(D817Y) performed as for Fig. 2a. (**e**,**f**) Subcellular localization of Kit. (**e**) Methanol-fixed HMC-1 or (**f**) PFA-fixed RBL-2H3 cells were double-stained with anti-Kit (green) and anti-cathepsin D (endolysosome marker; red), anti-LAMP1 (endolysosome marker; red), anti-calnexin (ER marker; red), or anti-GM130 (Golgi marker; blue). Magnified images of the boxed area are shown. Representative images of Kit-positive endolysosomes containing cathepsin D are shown. Bars, 5 μm. The graphs show the correlation coefficient (Pearson’s R) between Kit and organelle markers. Results are means±s.d. from 15 to 18 cells. Data were subjected to one-way ANOVA with Dunnett’s multiple comparison *post-hoc* test. **P<0.01, ***P<0.001. (**g**,**h**) Endocytosis of mutant Kit in HMC-1 and RBL-2H3 cells. (**g**) HMC-1 (left) or RBL-2H3 cells (right) treated with 1 μM PKC412 for 4 or 12 h, respectively. Cells were stained with anti-Kit (green) and anti-calnexin (ER marker; red). Insets show boxed areas at higher magnification. Bars, 10 μm. (**h**) HMC-1 cells treated with 1 μg ml^−1^ filipin or 0.45 M sucrose for 3 h to block endocytosis. Cells were stained with anti-Kit (cyan) and anti-p85 (red). Bars, 10 μm. Immunoblots for Kit, p85 and TfR are shown.

Next, we investigated whether the oncogenic role of Kit(D814Y) and its trafficking seen in RCM cells occur widely in neoplastic mast cells. The human and rat mast cell leukemia cell lines HMC-1 and RBL-2H3 endogenously express Kit with mutations in the kinase domain, these being Kit(D816V) and Kit(D817Y), respectively (Fig. 7a)^12^,^13^. In these lines, as with RCM, the kinase inhibitor PKC412 blocked Kit kinase activity and cell proliferation (Fig. 7b,c). Most mutant Kit was present as a complex-glycosylated form (Fig. 7d) that significantly co-localized with endolysosomal markers cathepsin D or LAMP1, rather than calnexin (ER) or GM130 (Golgi) (Fig. 7e,f). In HMC-1 cells, anti-Kit staining shows a pattern similar to that of expressed Kit(D814Y)-GFP but not to Kit(wt)-GFP (Supplementary Fig. 6a), confirming the endolysosomal Kit(D816V) staining.

We next examined Kit trafficking to endolysosomes in these cells. Figure 7g shows that PKC412 inhibited trafficking from the PM to endolysosomes, consistent with our findings on Kit(D814Y). Furthermore, for endocytosis, CME inhibition by sucrose reduced the protein levels of Kit but not of p85; in contrast, NCE inhibition by filipin did not affect the protein levels (Fig. 7h and Supplementary Fig. 6b). This suggests that in HMC-1 and RBL-2H3 cells, as in RCM cells, Kit also undergoes CME. Taken together, these results suggest that, in HMC-1 and RBL-2H3, Kit’s oncogenic role and intracellular trafficking are similar to those in RCM cells.

### Kit signalling occurs in distinct compartments in human and rat cells

**Figure 8:**
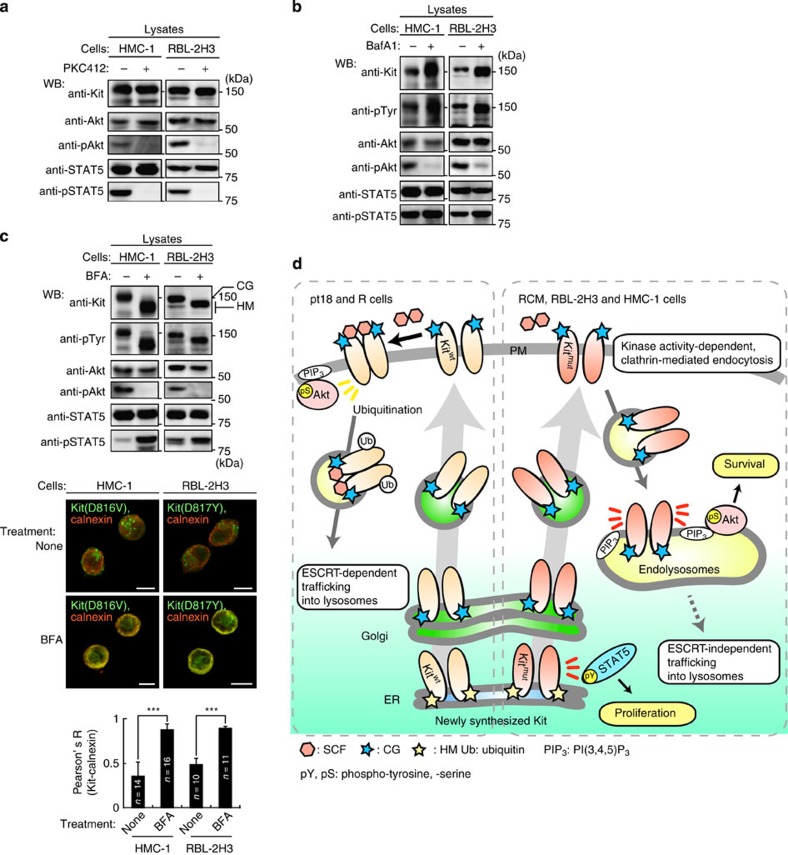
Oncogenic signalling by mutant Kit on endolysosomes and the ER in rat and human cells. (**a**) Constitutive activation of Akt and STAT5 by mutant Kit in HMC-1 and RBL-2H3 cells. Immunoblots, HMC-1 (left) or RBL-2H3 cells (right) treated with 1 μM PKC412 for 4 or 12 h, respectively. (**b**) Immunoblots, HMC-1 or RBL-2H3 cells cultured with 100 nM BafA1 for 24 h. (**c**) (Top) Immunoblots, HMC-1 or RBL-2H3 cells cultured with 5 μM BFA for 16 h. CG, complex-glycosylated form; HM, high mannose form. (Middle) Cells stained with anti-Kit(green) and anti-calnexin (ER marker; red). Bars, 10 μm. (Bottom) The graph shows the correlation coefficient (Pearson’s R) between Kit and calnexin. Results are means±s.d. from 10 to 16 cells. ***P<0.001, Student’s *t*-test. (**d**) Trafficking and signalling from normal Kit (left) and mutant Kit (right). Normal Kit-left panel; newly synthesized Kit traffics from ER, through the Golgi apparatus to the PM. After binding to SCF, Kit activates downstream pathways such as PI3K-Akt mainly at the PM. Kit then becomes endocytosed and ubiquitinated, resulting in ESCRT-dependent incorporation into lysosomes and rapid degradation. Mutant Kit-right panel; soon after synthesis, immature Kit is localized on the ER and activates STAT5. It then traffics to the PM along the secretory pathway, similar to normal Kit. After mutant Kit reaches the PM, it immediately undergoes clathrin-mediated endocytosis in a kinase activity-dependent manner. Kit then accumulates to endolysosomes but is not fully ubiquitinated, so is resistant to degradation. Unlike normal Kit, mutant Kit-PI3K activates Akt specifically on endolysosomes. Mutant Kit does not require ESCRT for incorporation into lysosomes.

Next, we examined the subcellular location for signalling in HMC-1 and RBL-2H3. As in RCM, oncogenic activation of Akt and STAT5 was also seen and was prevented by PKC412 (Fig. 8a and Supplementary Fig. 6c). Blockade of Kit trafficking to endolysosomes by BafA1 for 24 h also suppressed the activation of Akt but not of STAT5 (Fig. 8b and Supplementary Fig. 6d), indicating that endolysosomal localization of Kit is essential for Akt activation. Blockade of ER export of Kit by BFA for 16 h suppressed Akt activation, but enhanced STAT5 activation (Fig. 8c). Collectively, these results suggest that in HMC-1 and RBL-2H3 cells, as in RCM cells, partially glycosylated Kit aberrantly activates STAT5 on the ER.

## Discussion

In contrast to normal Kit, which signals from the PM, mutant oncogenic Kit signals from intracellular compartments (Fig. 8d). Newly synthesized, incompletely glycosylated mutant Kit initially localizes to the ER then activates STAT5. Subsequently, mutant Kit traffics to the PM through the Golgi along the secretory pathway and then immediately undergoes CME due to its kinase activity. It then accumulates in endolysosomes, but is not fully ubiquitinated. Mutant Kit is constitutively associated with PI3K, but the complex activates Akt only on the cytoplasmic surface of endolysosomes. In addition to previous reports^25^,^26^,^30^,^31^,^32^,^33^, our study shows that the oncogenic signalling from mutant Kit is spatially distinct from normal signalling. By comparing RCM with R cells, we believe that our results show the significance of the unusual Kit localization seen in neoplastic mast cells.

Compartment-dependent oncogenic signalling by constitutively active growth factor receptors has occasionally been reported before. In multiple myeloma cells, deregulated FGF receptor 3 mutants accumulate at the Golgi, and initiate Erk1/2 and STAT1/3 signalling^22^,^27^. Oncogenic gp130 activates STAT3 not only on the ER but also on endosomes in hepatocytes^59^. In acute myeloid leukemia, the oncogenic Flt3 mutant Flt3-ITD activates STAT5 only on the ER^25^,^26^,^27^, as shown here for mutant Kit. Kit(D816V) directly tyrosine-phosphorylates STAT proteins *in vitro*^21^, suggesting that STAT5 might act as a substrate for ER-localized mutant Kit. The Kit-STAT5 complex might escape from negative regulators such as protein tyrosine phosphatases on the ER but not on the Golgi, endosomes or the PM. The signalling platform may depend on the receptor and type of cell. These studies support our model that oncogenic Kit signalling occurs only on specific intracellular compartments in neoplastic mast cells. In leukaemia, mutant Kit participates in the PI3K-Akt pathway, in STAT5 activation, in the RhoA-Rho kinase pathway^47^ and in activation of Src-like adaptor protein^60^, and there is great interest in further understanding the spatial organization of this signalling.

Mutant Kit associates with PI3K throughout its intracellular trafficking, but only activates Akt when on endolysosomes. The PI3K product PI(3,4,5)P_3_, which is required for Akt activation, is believed to be generated solely at the PM^54^,^61^,^62^. However, PI(4,5)P_2_, a substrate of PI3K, is generated by phosphatidylinositide-4-phosphate-5-kinase on LAMP1-positive structures for proto-lysosome homeostasis^63^,^64^ and plays a role in endosome-to-lysosome trafficking^52^. The following observation is also relevant: when SCF binds Kit at the PM, Kit activates inositol-5-phosphatase, which dephosphorylates PI(3,4,5)P_3_, and so terminates Akt signalling^10^. The significance is that, unlike normal Kit, mutant Kit cannot activate Akt at the PM. Further studies will be required to understand the mechanism by which the Kit-PI3K complex activates Akt selectively on endolysosomes.

In this study, CME stabilized mutant Kit on endolysosomes. This agrees with previous data showing that CME is essential for sustained signalling from Met, TGFβR and EGFR^24^,^37^,^38^,^39^,^50^. However, mutant Kit was barely found in recycling endosomes, where the other receptors localize after ligand stimulation, and then recycle back to the PM. As very little SCF-bound normal Kit and mutant Kit recycle back to the PM^33^,^45^, CME must lead to at least two different pathways: the endolysosomal pathway leading to receptor destruction and the recycling endosome pathway, leading to receptor recycling. Ubiquitination of mutant Kit is insufficient for its rapid degradation. Defects in the tumour suppressor protein Cbl, an E3-ubiquitin ligase, also slow the degradation of Kit and Flt3, and prolong the activation of Akt, resulting in mastocytosis and myelo-proliferative disease^43^,^65^,^66^. When the CME pathway was blocked, mutant Kit was sorted into NCE and normally ubiquitinated. Neoplastic transformation might also involve an inappropriate sorting mechanism that recruits mutant Kit predominantly into CME not NCE. At present, we cannot explain why.

After SCF stimulation, Kit(wt) is transported into lysosomes in a manner dependent on ESCRT. This is consistent with previous reports that ESCRT transports ubiquitinated cargos^44^,^46^,^52^. ESCRT inhibition, however, does not cause accumulation of Kit(D814Y). Importantly, a recent study described that under-ubiquitinated cargos are incorporated into lysosomes in a manner independent of ESCRT^46^. Our results suggest that the mechanism of endosome-lysosome trafficking for mutant Kit is different from that for Kit(wt). Other mutant RTKs, such as EGFR(L858R)^23^, Met(D1246N)^24^ and Flt3(D835Y)^25^, might also escape from ESCRT-dependent trafficking.

Imatinib, a Kit inhibitor used as a drug, is efficacious in most patients with gastrointestinal stromal tumours harbouring Kit mutations in the juxta-membrane region^4^,^11^,^14^. However, imatinib is ineffective for most human mast cell tumours with Kit mutations in the kinase domain^20^,^29^,^67^. Moreover, during imatinib treatment of the gastrointestinal tumours, mutations often develop in the Kit kinase domain^14^. Imatinib-resistant gastrointestinal tumour cells depend on activation of the PI3K-Akt pathway by Kit mutants for their proliferation, and so resemble mast cells^68^.

In this study, we showed that, in mast cells, mutant Kit trafficking to endolysosomes is critical for the activation of Akt. When acute myeloid leukemia cells with constitutively active Flt3 mutants, such as Flt3-ITD and Flt3(D835Y), are treated with statins (HMG-CoA reductase inhibitors), this reduces Akt activation by blocking receptor trafficking towards the PM^25^. Thus, Kit trafficking could be a new therapeutic target for mast cell tumours, and for imatinib-resistant gastrointestinal tumours. Combined therapy with an antibody and a kinase inhibitor seems attractive. As PKC412 treatment causes mutant Kit to remain at the PM through blocking the Kit kinase activity, it might enhance the activity of anti-Kit antibody^69^. Lapatinib, an EGFR kinase inhibitor, enhances the cytotoxic activity of trastuzumab, an anti-ErbB2 antibody *in vivo*^70^.

In conclusion, we show that compartment-dependent oncogenic Kit signalling is necessary for neoplastic mast cell proliferation. These findings provide new insights into the pathogenic role of Kit in neoplastic mast cell disorders. Improper trafficking and aberrant signalling are frequent features of constitutively active growth factor receptors, for which these data will shed light on the significance of the spatial organization of this oncogenic signalling.

## Methods

### Cells and stimulation

RCM (R cell, mutant Kit) and R cells were established from splenocytes of DO11.10 mice by repeated stimulation with ovalbumin peptides *in vitro*. The cell lines exhibited mast cell-like surface phenotype, c-Kit^+^ FcεRI^+^, and mast cell-like expression profiles of proteases. Moreover, RCM and R cells can secrete biologically active product on stimulation *in vitro* and *in vivo*. They do not express SCF. We were unable to find *Kit(wt)* by cDNA sequencing in RCM cells. For culture of R cells, we used culture supernatants from T-cell lines stimulated with an anti-T cell receptor antibody as a cytokine cocktail. R cells were cultured in 0.25% cytokine cocktail. RCM cells proliferated without the cocktail and developed tumours *in vivo*. HMC-1.2 (referred to henceforth as HMC-1), RBL-2H3 and pt18 cells were fro m Dr Hirohisa Saito and Dr Kenji Matsumoto (National Center for Child Health and Development), Dr Ko Okumura (Juntendo University) and Dr Ryo Goitsuka (Tokyo University of Science), respectively. These cells and RCM cells were cultured at 37 °C in RPMI1640 medium supplemented with 10% fetal calf serum (FCS), penicillin, streptomycin and 50 μM 2-mercaptoethanol. HMC-1 cells were grown in suspension at 37 °C in α-MEM containing 10% FCS, penicillin and streptomycin. For stimulation, cells were starved for at least 3 h and then treated with 50 ng ml^−1^ recombinant mouse SCF (PeproTech).

### Cell proliferation assay

Cells were cultured with inhibitors or cytokines for at least 16 h and then treated with [^3^H]-thymidine deoxyribonucleotide (TdR) for 8 h. Cell proliferation was evaluated by incorporation of [^3^H]-TdR.

### Chemicals

PKC412 (Santa Cruz Biotechnology), cytochalasin D (Sigma-Aldrich), tunicamycin (Sigma-Aldrich), cycloheximide (Sigma-Aldrich), filipin (Sigma-Aldrich), Akt inhibitor VIII (Calbiochem), STAT5 inhibitor (Calbiochem), LY294002 (Calbiochem), U0126 (Calbiochem) and pitstop2 (Abcam) were dissolved in dimethyl sulfoxide. Bafilomycin A1 (Sigma-Aldrich), brefeldin A (Sigma-Aldrich) and monensin (Biomol) were dissolved in ethanol.

### Antibodies

The following antibodies were purchased: c-Kit (M-14), STAT3 (C-20), STAT5 (C-17), Erk2 (K-23), Jak2 (C-20), cathepsin D (H-75), CD63 (H-93), actin (I-19) and CD28 (H-193) from Santa Cruz Biotechnology; Kit[pTyr719], Akt, Akt[pSer473], STAT5[pTyr694] (D47E7), cleaved caspase-3 and Erk[pThr202/pTyr204] (E10) from Cell Signaling Technology; TGN46, EEA1, Rab11, Tsg101 and TfR from Abcam; calnexin and ubiquitin (FK2) from Enzo; GM130 (35) and AP2α (8) from BD Transduction Laboratories; p85 from Millipore and LAMP1 from Sigma-Aldrich. Anti-phosphotyrosine antibody (4G10) was kindly provided by Dr Toshinori Nakayama (Chiba University). Alexa-fluor 488 anti-Kit (AF488-anti-Kit; 2B8; Biolegend) was used for the experiments in Figs 2b and 5k. The list of antibodies with source and conditions of immunoblotting and immunofluorescence is shown in Supplementary Table 1. HRP-labelled anti-mouse Ig, anti-rabbit Ig and anti-goat Ig secondary antibodies were purchased from The Jackson Laboratory. AF488-anti-goat IgG, AF568-anti-rabbit IgG, AF647-anti-goat IgG and AF647-anti-mouse secondary antibodies were obtained from Molecular Probes.

### Plasmids

Mouse cDNAs encoding Kit(wt) or Kit(D814Y) were generated by reverse transcription polymerase chain reaction from R or RCM cells, respectively. After deletion of the termination codon, they were fused with GFP from pEGFP-N1 (Clontech). For protein expression, constructs were subcloned into the Xho I and Not I sites of pBCMGS (from Dr Hajime Karasuyama, Tokyo Medical and Dental University). PH-GFP or PH(R25C)-GFP subcloned into pEGFP-N1 (ref. 54) were from Dr Tamás Balla (National Institutes of Health) through Addgene. The human p85 N-terminal SH2 domain (325–430) or C-terminal SH2 domain (614–721) subcloned into pGEX-4T-2 (GE Healthcare Life Sciences) were from Dr Masayuki Oda (Kyoto Prefectural University).

### Gene silencing with siRNAs

For silencing *Kit(D814Y)*, *STAT5B*, *AP2α* or *Tsg101*, siRNA duplexes were purchased from Sigma-Aldrich (Kit1: 5′- GAAGGAUUAUGUCAAAUCUTT -3′, Kit2: 5′- GACAUGAAGCCUGGCGUUUTT -3′, STAT5-1: 5′- GAAUUUGCCAGGACGGAAUTT -3′, STAT5-2: 5′- GGAAUUACACUUUCUGGCATT -3′, Tsg1: 5′- GACACATACCCATATAACCCC -3′, Tsg2: 5′- ACCCGCTTAGATCAAGAAGTA -3′, AP2α-1: 5′- GCAAAGAGGCTGAGATCAAGA -3′, AP2α-2: 5′- GGGTTATGCTGCCAAGACAGT -3′). The control siRNA duplex was purchased from Sigma-Aldrich (Mission negative control SIC-001). No STAT5A was observed in RCM cells.

### Transfection

For protein expression or knockdown, cells were transfected by using a Gene Pulser II electroporation system (Bio-Rad Laboratory) and cultured for at least 20 h. Cells expressing Kit(D814Y)-GFP, PH-GFP or PH(R25C)-GFP were selected in 1 mg ml^−1^ G-418.

### Immunofluorescence

Cells were fixed with 4% PFA for 20 min at room temperature, or with methanol for 10 min at −20 °C, then cyto-centrifuged onto coverslips. Fixed cells were permeabilized and blocked for 30 min in PBS supplemented with 0.1% saponin and 3% bovine serum albumin, and then incubated with a primary and a secondary antibody for 1 h each. To stain with anti-Akt(pSer473) (Cell Signaling Technology; 193H12), 10% skimmed milk was used for blocking. After washing with PBS, cells were mounted with Fluomount (DiagnosticBioSystems). For staining endocytic compartments, cells were incubated for 1 h with 5 μg ml^−1^ AF647-CTXB or 1 mg ml^−1^ AF647-dextran (Molecular Probes). Confocal images were obtained by a Fluoview FV10i laser scanning microscope with an x60 1.20 N.A. water-immersion objective (Olympus). Composite figures were prepared with Photoshop elements 10 and Illustrator CS6 software (Adobe). Pearson’s correlation coefficients (Pearson’s R) were calculated with NIH ImageJ Version 1.48v software.

### Immunoprecipitation and western blotting

Lysates from RCM, R, pt18, HMC-1 or RBL-2H3 cells were prepared in NP-40 lysis buffer (50 mM HEPES, pH 7.4, 10% glycerol, 1% NP-40, 4 mM EDTA, 100 mM NaF, 1 μg ml^−1^ aprotinin, 1 μg ml^−1^ leupeptin, 1 μg ml^−1^ pepstatin A, 1 mM PMSF and 1 mM Na_3_VO_4_). Kit from 2 × 10^6^ cells was immunoprecipitated in each assay. Immunoprecipitation was performed at 4 °C for 5 h using protein-G pre-coated with 1 μg of antibody. Samples were dissolved in SDS–polyacrylamide gel electrophoresis (SDS–PAGE) sample buffer, and subjected to SDS–PAGE and electro-transferred onto PVDF membranes. Immunodetection was performed by ECL (PerkinElmer). Sequential re-probing of membranes was performed after the complete removal of primary and secondary antibodies in stripping buffer (Thermo Scientific), or inactivation of peroxidase by 0.1% NaN_3_. Results were analysed with an LAS-3000 image analyzer with Science Lab software (Fujifilm Co.) or with a c-Digit imaging system with Image Studio Digit software (Licor Biosciences). Uncropped versions of the most important blots are shown in Supplementary Figs 7–11.

### Purification of endolysosomal vesicles

Homogenates from RCM or R cells were prepared by re-suspending in hypotonic buffer (20 mM HEPES pH 7.4, 1 mM MgCl_2_, 4 mM NaF, 10 mM EDTA, 0.01% NP-40, 1 μg ml^−1^ aprotinin, 1 μg ml^−1^ leupeptin, 1 μg ml^−1^ pepstatin A, 1 mM PMSF and 1 mM Na_3_VO_4_), and subsequent Dounce homogenization (20 strokes). The suspension was pre-cleared by centrifuging at 1,000 × *g* for 10 min at 4 °C. Endolysosomes were immunoprecipitated with anti-LAMP1-coated protein-G Dynabeads (Veritas) and subjected to immunoblotting. Rabbit anti-CD28 antibody was used for control IgG. Immunoprecipitation was performed at 4 °C for 12 h using 1.5 μg of anti-LAMP1 or anti-CD28. For each assay, 5 × 10^6^ cells were used.

### GST-pulldown assay

GST-fusion proteins were expressed in the *E. coli* BL-21 strain on incubation with 0.5 mM IPTG at 22 °C for 12 h. The bacteria were lysed by sonication in RIPA buffer (50 mM HEPES, pH 7.4, 10% glycerol, 0.1% SDS, 0.25% sodium deoxycholate, 1% NP-40, 4 mM EDTA, 100 mM NaF, 1 μg ml^−1^ aprotinin, 1 μg ml^−1^ leupeptin, 1 μg ml^−1^ pepstatin A, 1 mM PMSF). GST-fusion proteins were collected on glutathione-Sepharose beads from RIPA lysates and washed four times with RIPA buffer. Pull-down assays were performed at 4 °C for 5 h in NP-40 lysates prepared from RCM or R cells. Kit from 1 × 10^6^ cells was pulled down in each assay. After extensively washing with NP-40 lysis buffer, the bead pellets were analysed by SDS–PAGE and immunoblotted with an anti-Kit antibody.

### Analysis of protein glycosylation

Following the manufacturer’s instructions (New England Biolabs), NP-40 cell lysates were treated with endoglycosidase H or peptide-N-glycosidase F for 1 h at 37 °C. The reactions were stopped with SDS–PAGE sample buffer, products were resolved by SDS–PAGE and immunoblotted with an anti-Kit antibody.

## Author contributions

Y.O. conceived, designed, performed and analysed data from all experiments, and wrote the paper. S.T. and E.W. characterized R cells as mast-like cells *in vivo* and *in vitro* by flow cytometry, histo-cytochemical staining, electron microscopy, proliferation assays and microarray analyses, and edited the manuscript. S.S. and S.O performed immunoblotting, immunoprecipitation assays, *in vitro* GST-pulldown assays and RNA interference experiments. H.E. advised on the design of the *in vitro* experiments and edited the manuscript. R.A. conceived and supervised the project, analysed data and wrote the manuscript.

## Additional information

**How to cite this article:** Obata, Y. *et al*. Oncogenic Kit signals on endolysosomes and endoplasmic reticulum are essential for neoplastic mast cell proliferation. *Nat. Commun.* 5:5715 doi: 10.1038/ncomms6715 (2014).

## Supplementary Material

Supplementary Information Supplementary Figures 1-11 and Supplementary Table 1
