# Supplementary material for: Oncogenic Kit signals on endolysosomes and endoplasmic reticulum are essential for neoplastic mast cell proliferation
Source: Nat Commun. 2014 Dec 10;5:5715. doi: 10.1038/ncomms6715 (PMC4284665; doi:10.1038/ncomms6715)
Supplement: Supplementary Information — Supplementary Figures 1-11 and Supplementary Table 1 [file ncomms6715-s1.pdf]

Supplementary Information

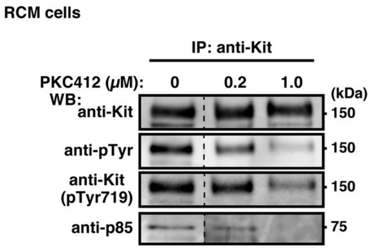

Supplementary Figure 1 l Inhibition of autophosphorylation of Kit(D814Y) by PKC412. RCM cells treated with 0, 0.2, or 1 μM PKC412 were cultured for 24 h, lysed, immunoprecipitated, and anti-Kit immunoprecipitates were immunoblotted.

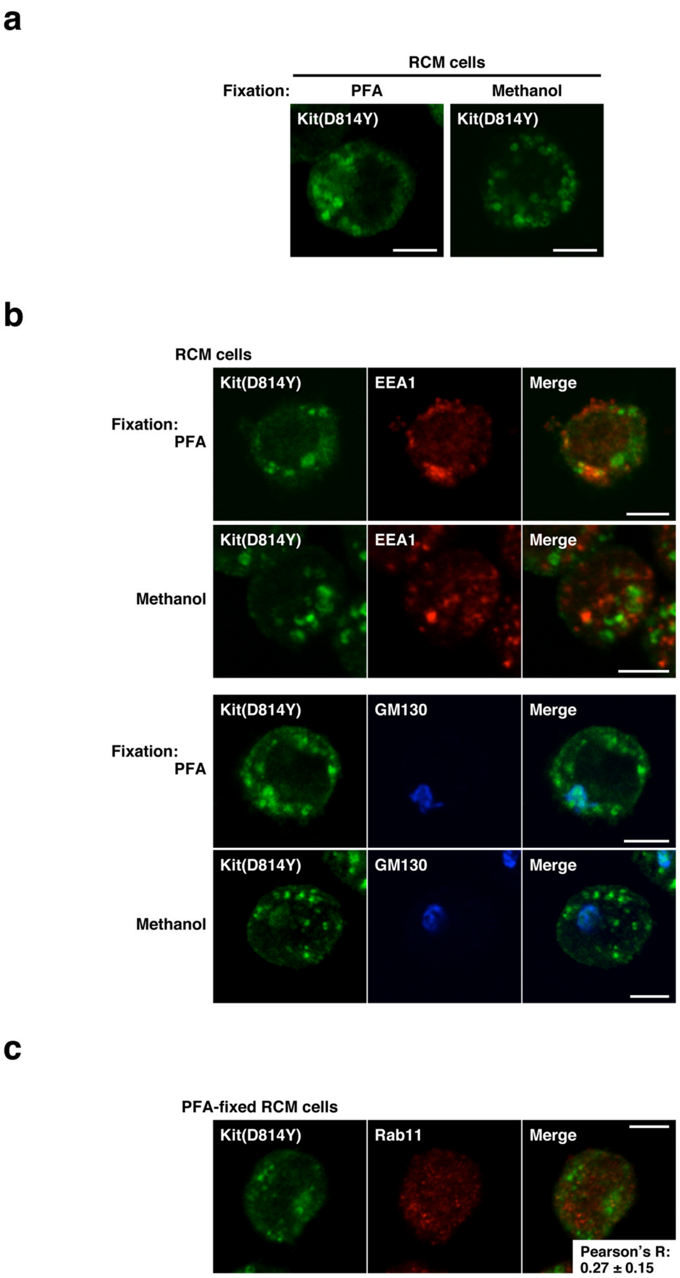

Supplementary Figure 2 l Subcellular localization of Kit(D814Y) in RCM cells. (**a**,**b**) RCM cells were fixed with PFA (left) or methanol (right) and stained with anti-Kit (green) plus anti-EEA1 (endosome marker, red) or anti-GM130 (Golgi marker, blue). Bars, 5 µm. (**c**) RCM cells were doubly stained with anti-Kit (green) and anti-Rab11 (recycling endosome marker, red). Pearson's correlation coefficient (Pearson's R) between Kit(D814Y) and Rab11 is shown. Results are means ± s.d. (n = 5). Bar, 5 µm.

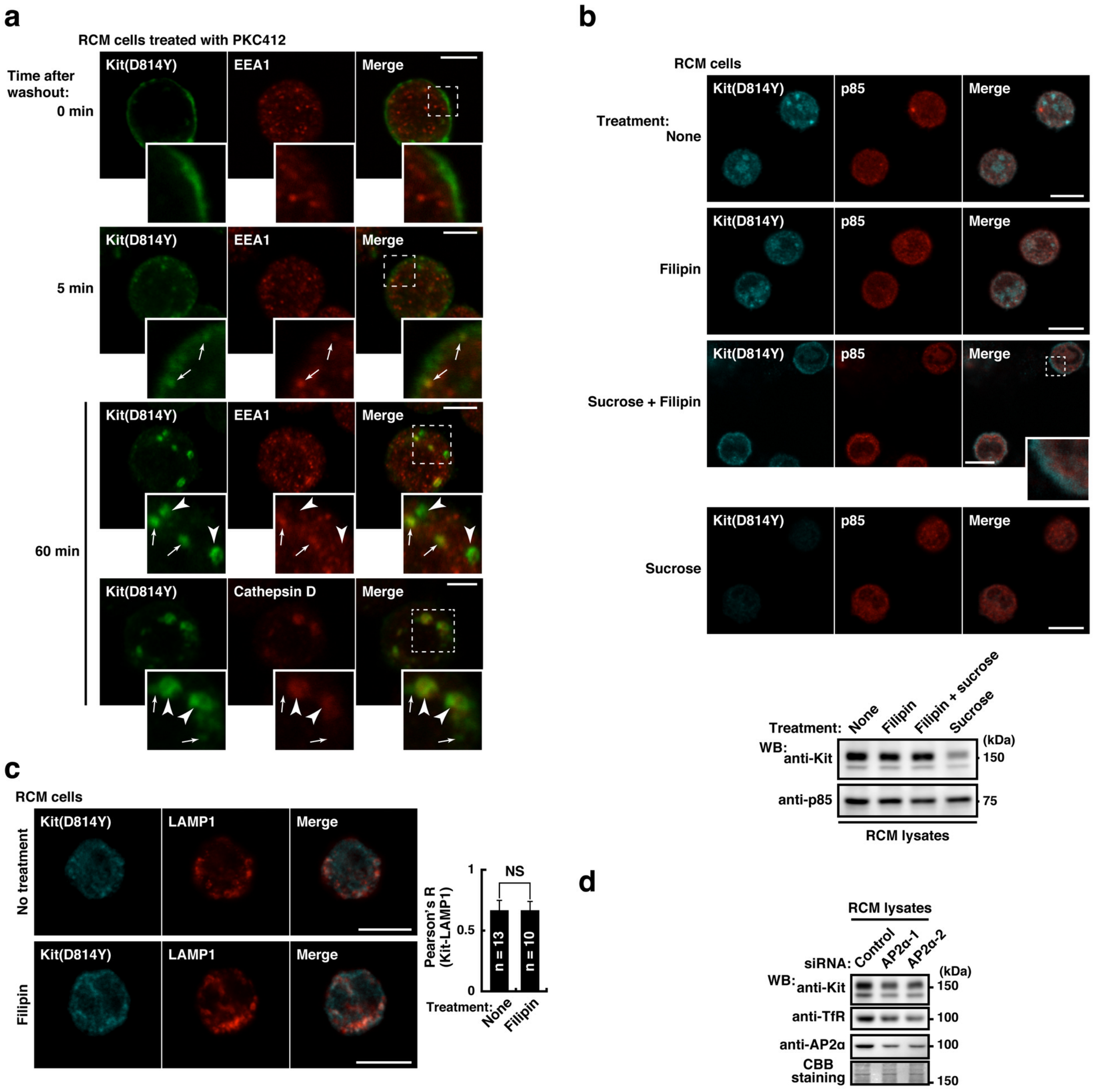

Supplementary Figure 3 | Trafficking of Kit(D814Y) from the plasma membrane to endolysosomes via clathrin-mediated endocytosis. (**a**) RCM cells were treated with 1 μM PKC412 (inhibitor of Kit tyrosine kinase) for 24 h. After washout of PKC412, cells were fixed with methanol when indicated and double-stained with anti-Kit (green) in conjunction with anti-EEA1 (endosome marker, red) or anti-cathepsin D (endolysosome marker, red). Arrows and arrowheads indicate early endosomes and endolysosomes, respectively. Insets show the magnified images of the boxed area. Bars, 5 μm. (**b**) Effect of sucrose and filipin on endocytosis of Kit(D814Y). RCM cells were treated with 0.45 M sucrose and/or 1 μg ml^{-1} filipin. After 3 h, cells were stained with anti-Kit (cyan) and anti-p85 (a cytosolic protein, red). Bars, 10 μm. Immunoblots are shown. (**c**) Effect of filipin on Kit's localization. RCM cells were treated with 1 μg ml^{-1} filipin and stained with anti-Kit (cyan) and anti-LAMP1 (endolysosome marker, red). Bars, 10 μm. The graph shows Pearson's R correlation coefficient between Kit(D814Y) and LAMP1. Results are means ± s.d. (n = 13 or 10). NS, not significant, Student's t-test. (**i**) RCM cells were transfected with AP2α siRNAs (AP2α-1 or AP2α-2) and cultured for 30 h. Lysates were immunoblotted. Total protein levels were confirmed by Coomassie staining.

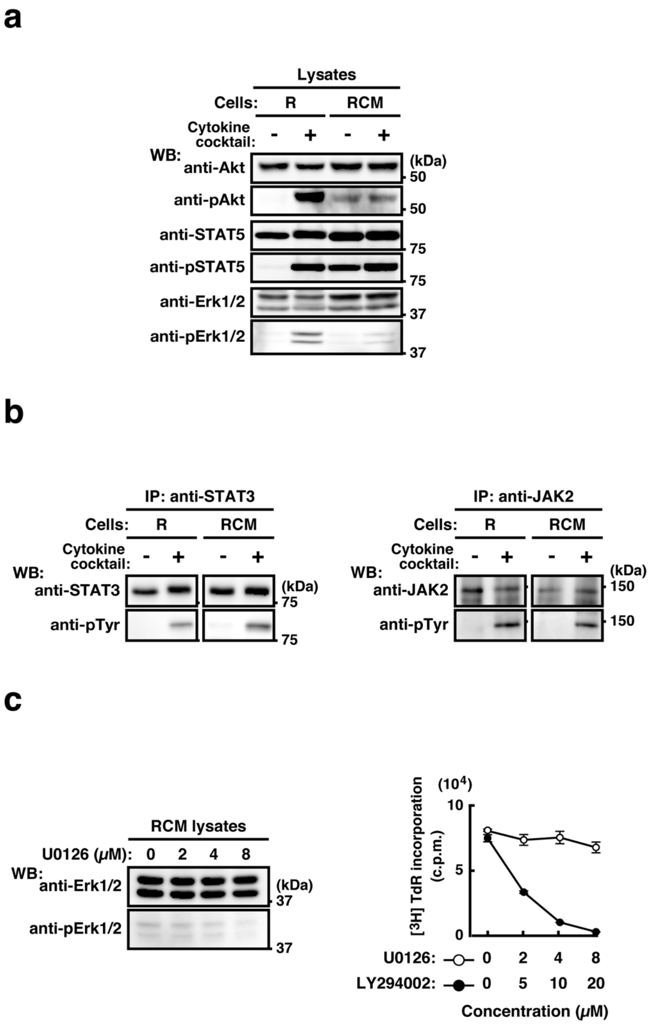

Supplementary Figure 4 | Constitutive activation of Akt and STAT5 in RCM cells. (**a**,**b**) Starved R and RCM cells were treated with the cytokine cocktail for 5 min. (**a**) Lysates were immunoblotted with the indicated antibody. (**b**) Immunoblots of anti-STAT3 immunoprecipitates (left) or anti-JAK2 immunoprecipitates (right). (**c**) Immunoblots of lysates from RCM cells treated with U0126 (inhibitor of MEK). The graph shows [^{3}H]-thymidine incorporation into RCM cells at the indicated concentrations of LY294002 (inhibitor of PI3K; filled circles) or U0126 (open circles). Results (c.p.m.) show means ± s.d. (n = 3). NB: U0126 did not affect the proliferation of RCM cells, compared with LY294002.

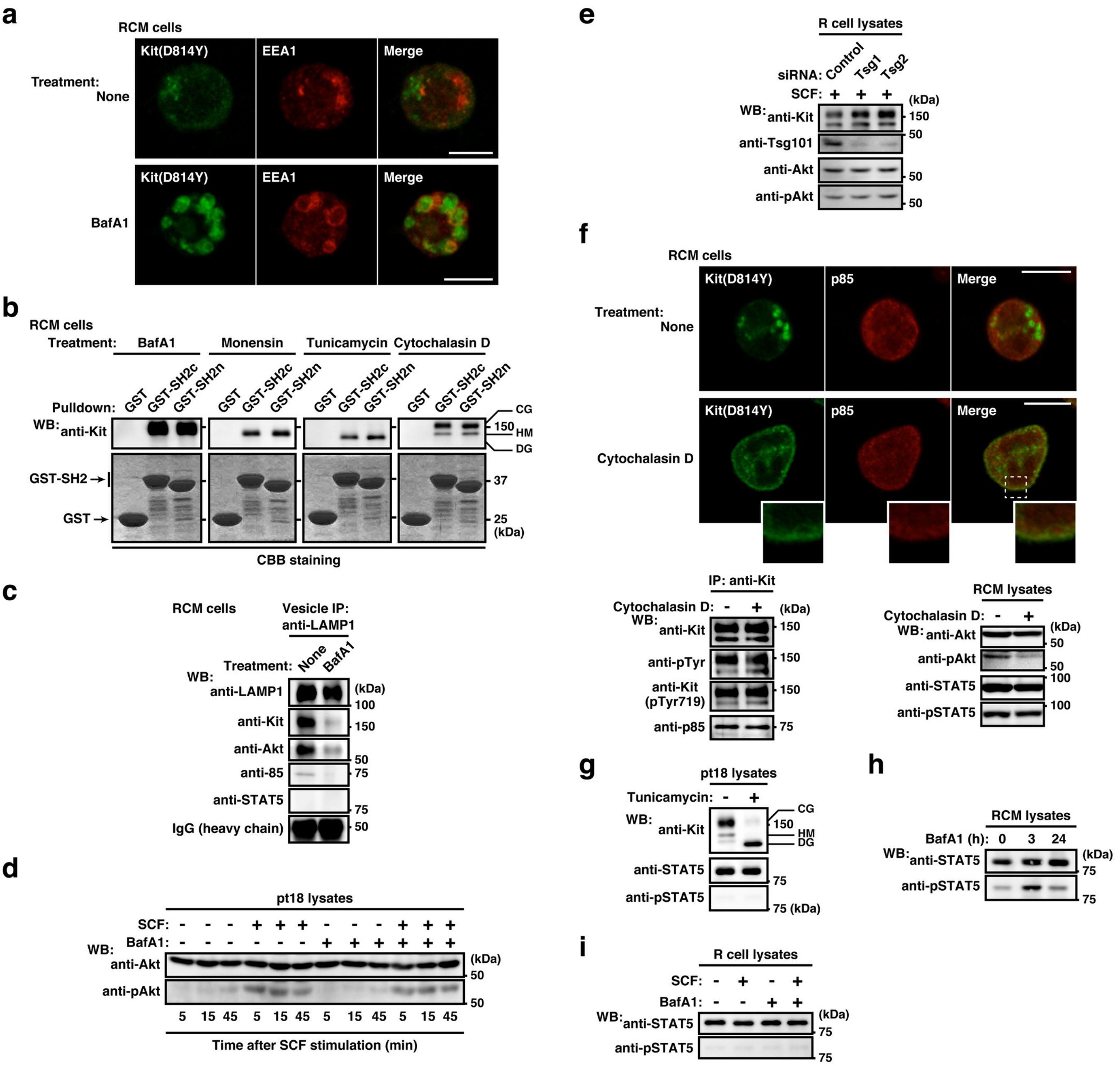

Supplementary Figure 5 | Inhibition of Kit(D814Y) trafficking to endolysosomes. (**a**) RCM cells were cultured with 100 nM BafA1 (blocks endosomal trafficking) for 24 h. Cells were stained with anti-Kit (green) and anti-EEA1 (endosome marker; red). Bars, 10 µm. (**b**) Lysates from RCM cells treated with the indicated inhibitors for more than 24 h were used for pulldown experiments with GST-p85c or GST-p85n, and amounts of Kit(D814Y) pulled down was assayed by immunoblotting with anti-Kit antibody. GST proteins were visualized by Coomassie staining. (**c**) RCM cells were cultured with 100 nM BafA1 for 24 h. Endolysosomes were immunoprecipitated with anti-LAMP1 then immunoblotted. (**d**) After 6-h treatment with 100 nM BafA1, pt18 cells were stimulated with 50 ng ml^{-1} SCF plus BafA1 then immunoblotted.
(**e**) Immunoblots, Tsg101-knocked down R cells treated with 50 ng ml^{-1} SCF for 30 min. (**f**) RCM cells were treated with 500 nM cytochalasin D (inhibits endocytosis) for 48 h. Cells were stained with anti-Kit (green) and anti-p85 (red). Inset shows the boxed area magnified. Bars, 10 µm. Immunoblots of anti-Kit immunoprecipitates (left) and lysates (right) are shown. (**g**) Immunoblots of anti-Kit immunoprecipitates and lysates from pt18 cells, after 12-h treatment with 1 µg ml^{-1} tunicamycin. CG, complex-glycosylated form; HM, high mannose form; DG, deglycosylated form. (**h**) RCM cells were treated with 100 nM BafA1 as indicated, then immunoblotted. (**i**) After 3-h treatment with 100 nM BafA1, R cells were stimulated with 50 ng ml^{-1} SCF for 30 min plus BafA1 then immunoblotted.

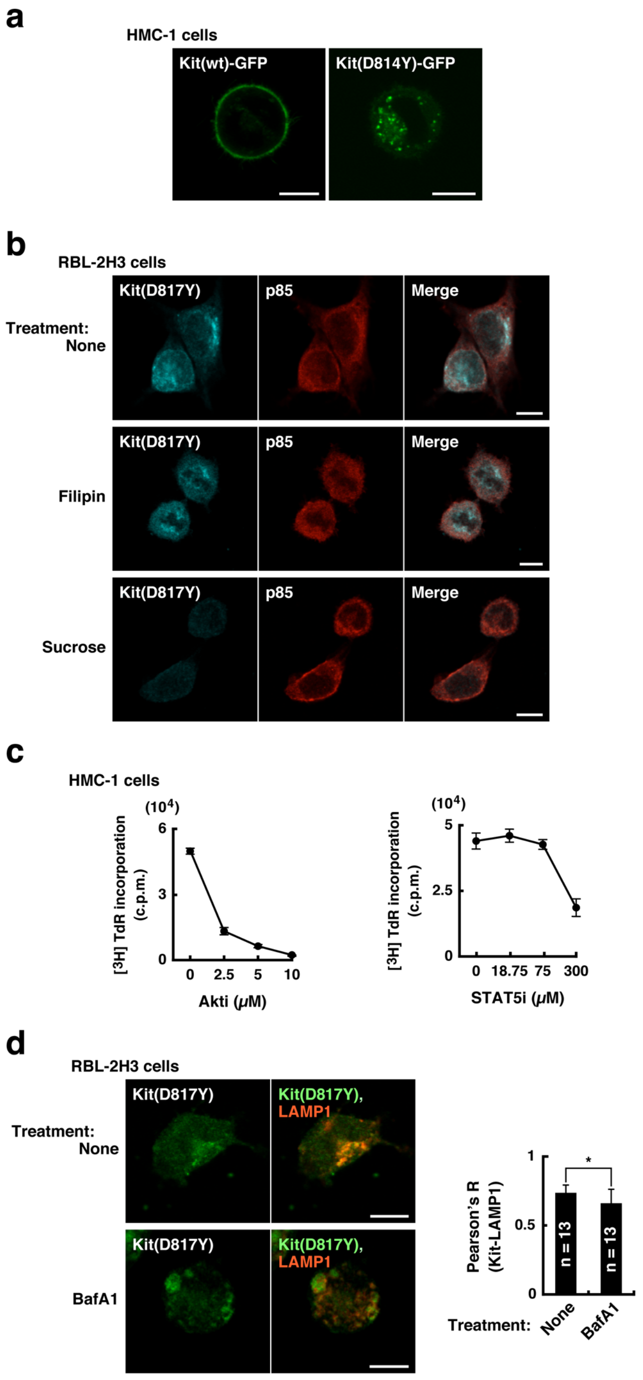

Figure 6 | Mutant Kit in HMC-1 and RBL-2H3 cells. (**a**) HMC-1 cells were transfected with Kit(wt)-GFP or Kit(D814Y)-GFP. Expressed proteins were visualized by GFP fluorescence. Bars, 10 μm. (**b**) RBL-2H3 cells were treated with 0.45 M sucrose or 1 μg ml^{-1} filipin for 3 h to block endocytosis. Cells were stained with anti-Kit (cyan) and anti-p85 (red). Bars, 10 μm. (**c**) [^{3}H]-thymidine incorporation into HMC-1 cells at the indicated concentrations of Akti (left) or STAT5i (right). Results (c.p.m.) show means ± s.d. (n = 3). (**d**) RBL-2H3 cells were cultured with 100 nM BafA1 for 24 h. Cells were stained with anti-Kit (green) and anti-LAMP1 (endolysosome marker; red). Bars, 10 μm. The graph shows Pearson's R correlation coefficient between Kit(D817Y) and LAMP1. *P < 0.05, Student's t-test.

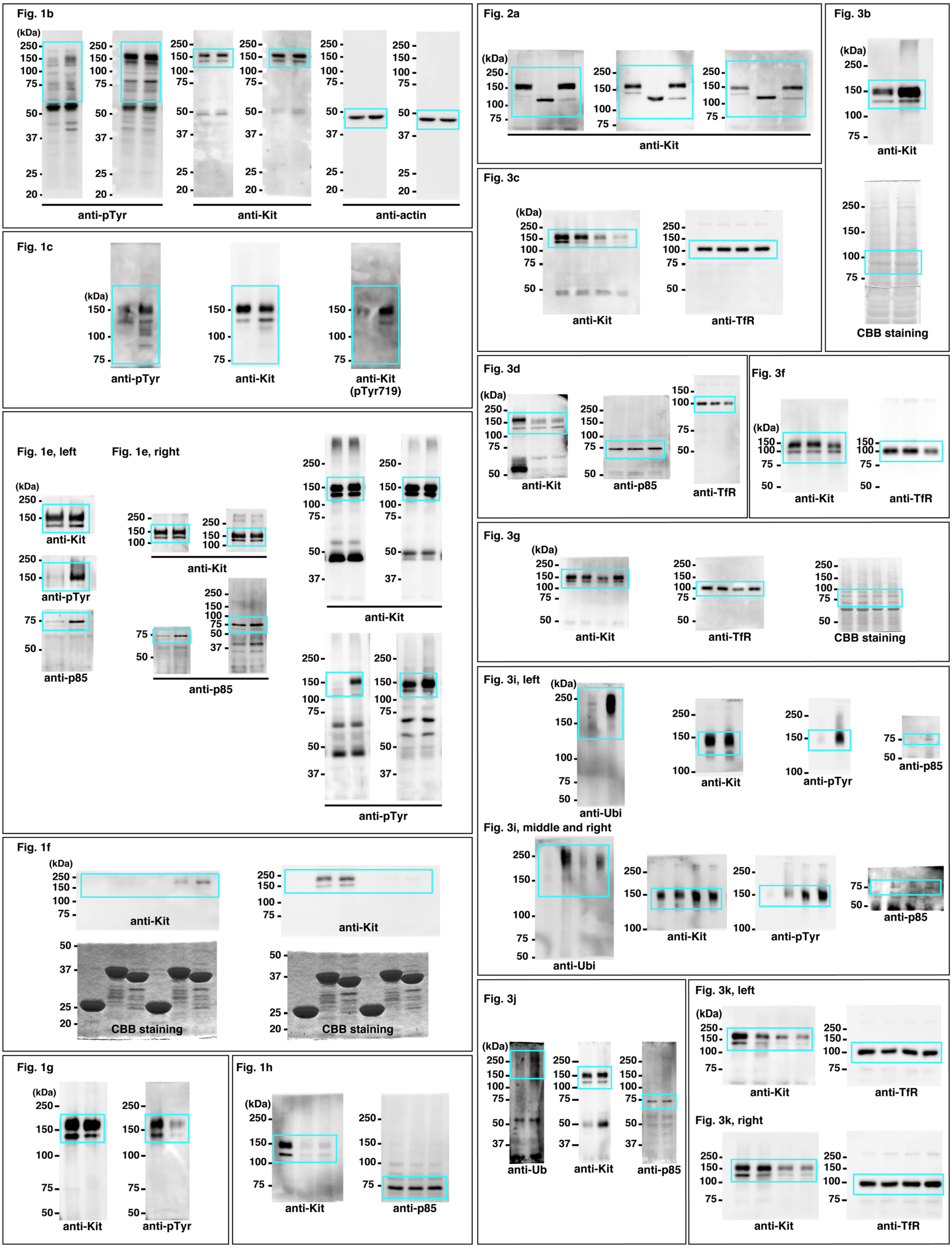

Supplementary Figure 7 | Uncropped versions of immunoblots referring to Figs 1b-3k.

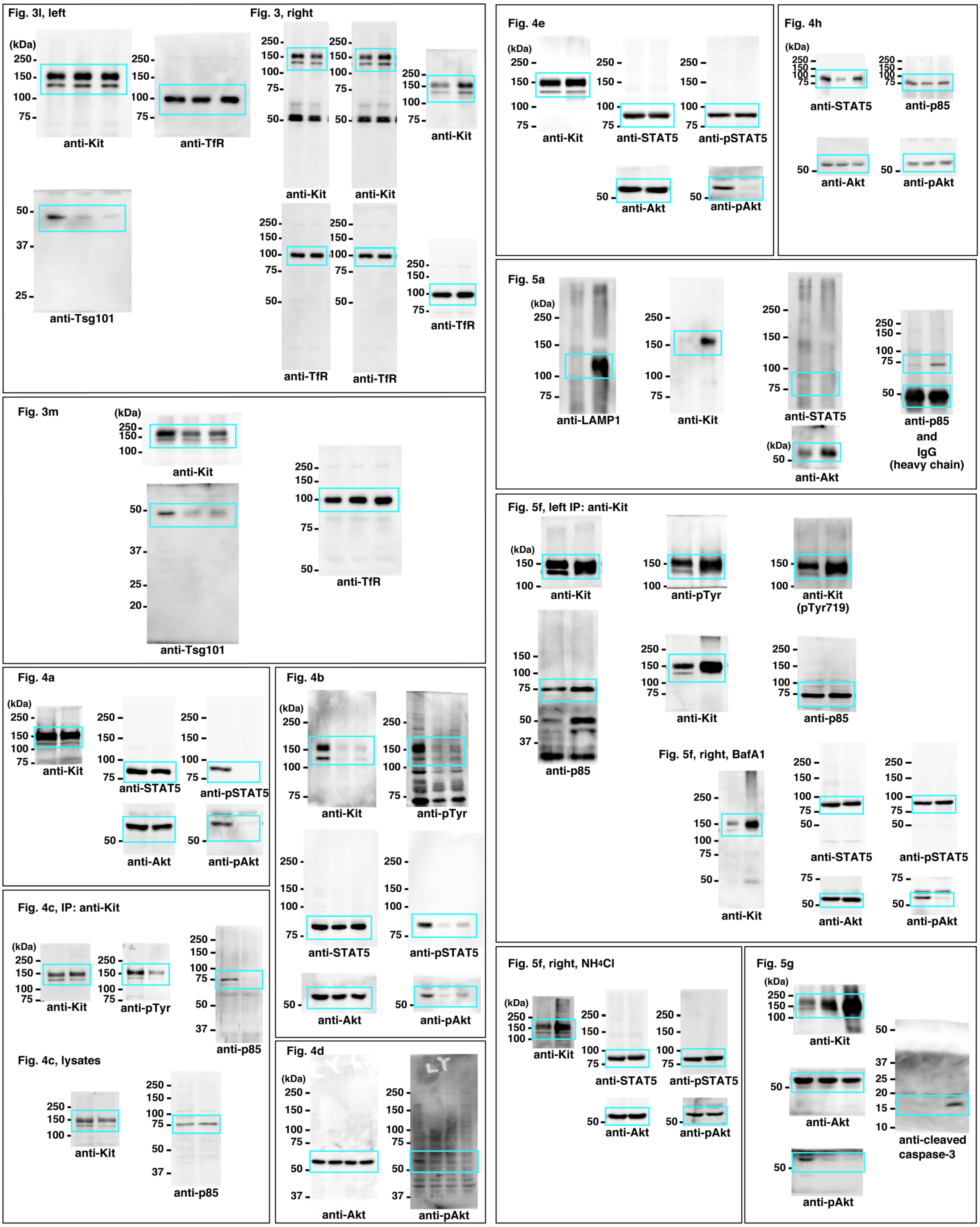

Supplementary Figure 8 l Uncropped versions of immunoblots referring to Figs 3l-5g.

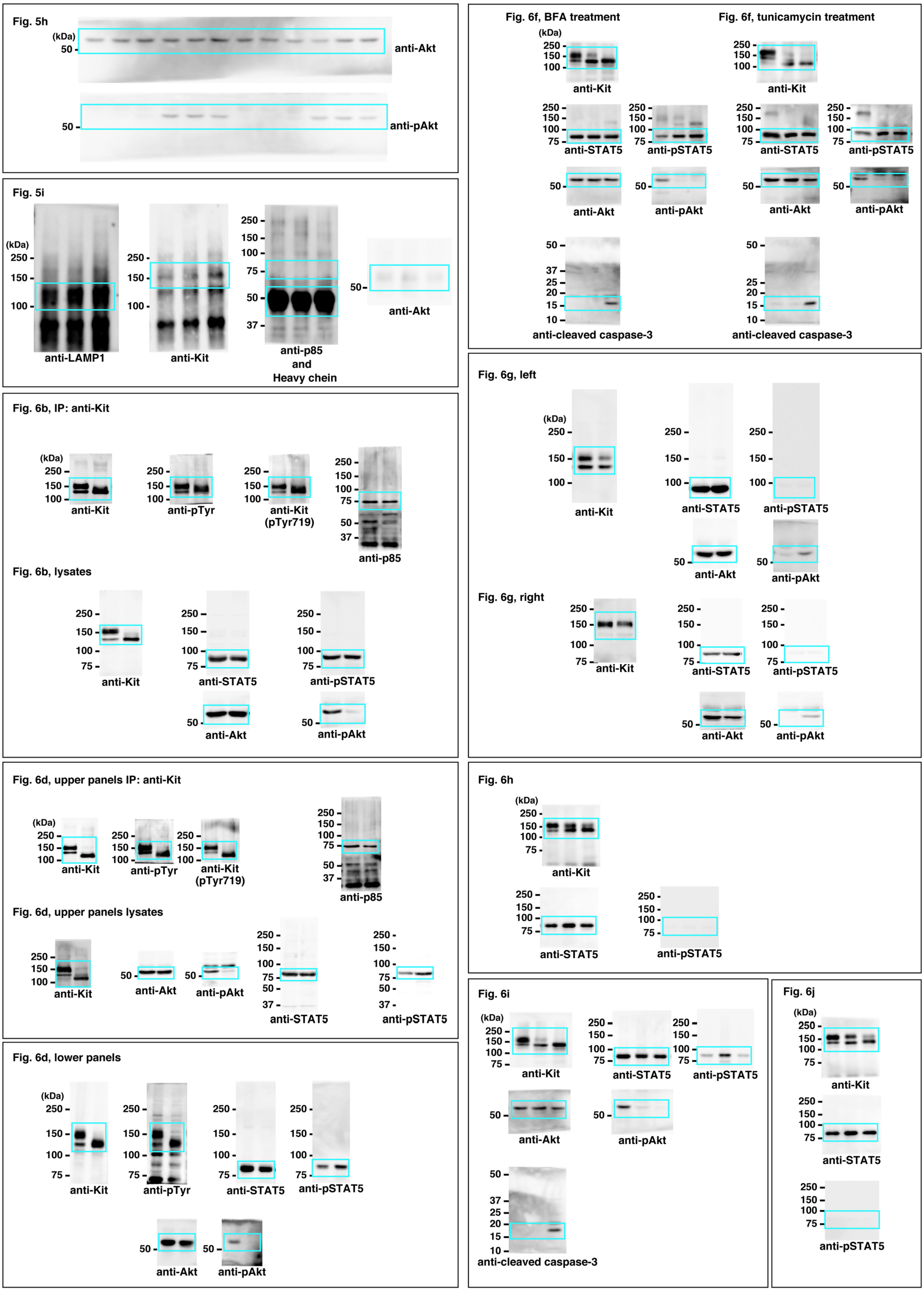

Supplementary Figure 9 l Uncropped versions of immunoblots referring to Figs 5h-6j.

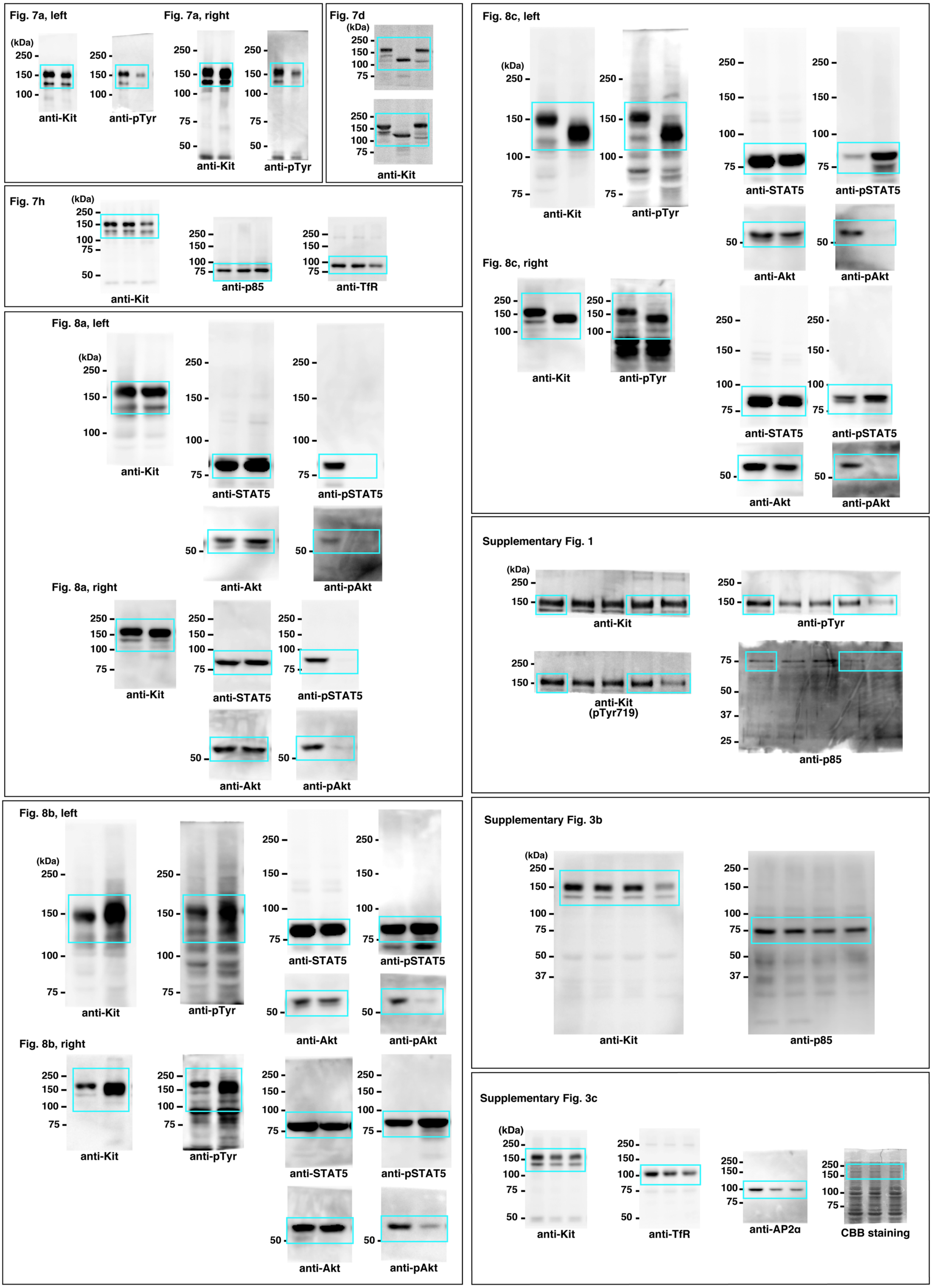

Supplementary Figure 10 | Uncropped versions of immunoblots referring to Figs 7a-8c and Supplementary Figs 1, 3b, and 3c.

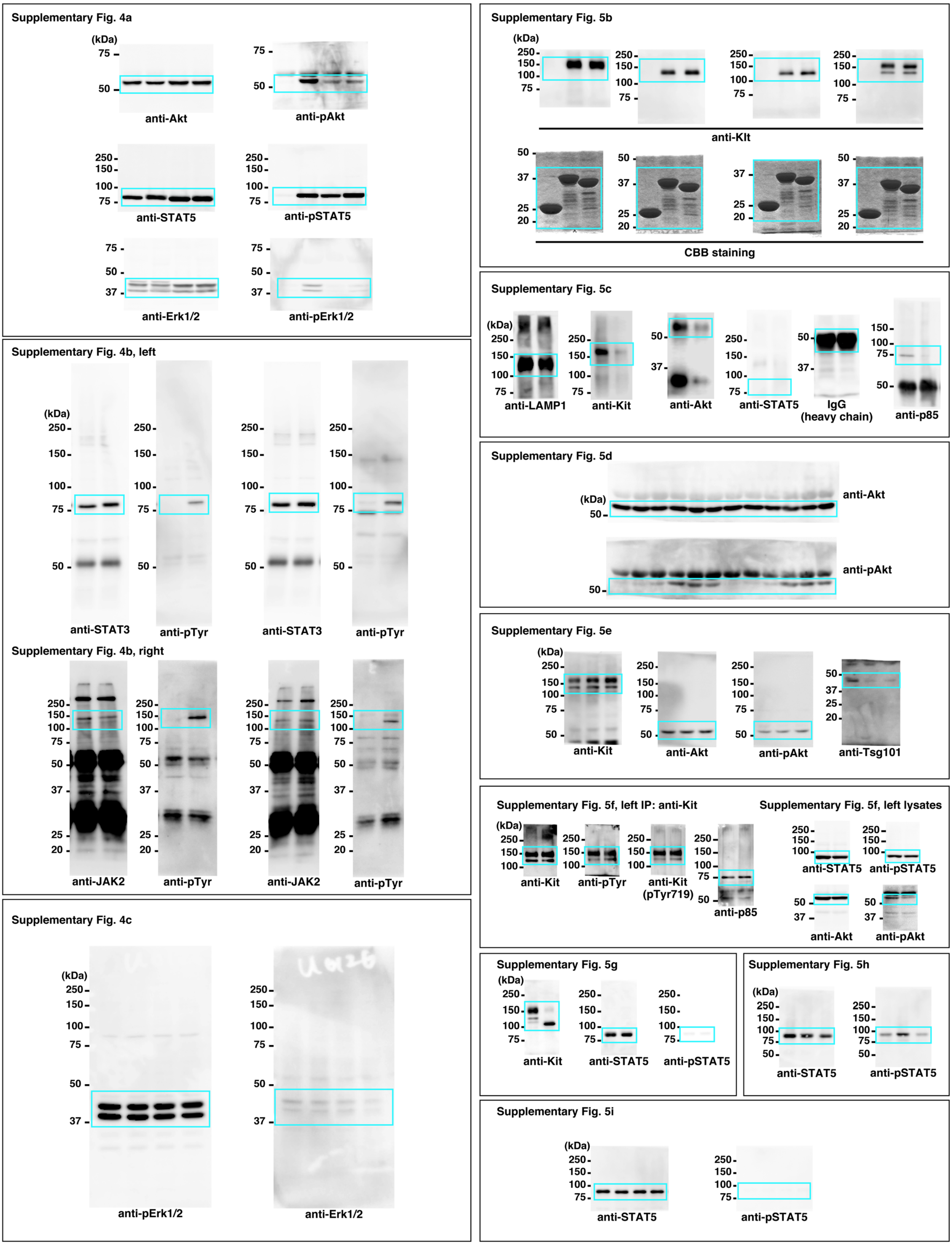

Supplementary Figure 11 | Uncropped versions of immunoblots referring to Supplementary Figs 4a-5i.

Supplementary Table 1. List of antibodies

Antibody	Clone/catalog #	Distribution source	WB	IF
Actin	I-19	Santa Cruz Biotechnology	1/500	-
Akt	#9272	Cell Signaling Technology	1/1000	-
Akt	40D4	Cell Signaling Technology	1/1000	-
Akt[pSer473]	#9271	Cell Signaling Technology	1/1000	-
Akt[pSer473]*	193H12	Cell Signaling Technology	-	1/100
AP2α	8	BD Transduction Laboratories	1/1000	-
Calnexin	ADI-SPA-860	Enzo	-	1/250
Cathepsin D	H-75	Santa Cruz Biotechnology	-	1/100
CD28	H-193	Santa Cruz Biotechnology	-	-
CD63	H-93	Santa Cruz Biotechnology	-	1/100
c-Kit	M-14	Santa Cruz Biotechnology	1/1000	1/250
c-Kit	2B8	Biolegend	-	1/100
c-Kit[pTyr719]	#3391	Cell Signaling Technology	1/1000	-
Cleaved caspase-3	#9661	Cell Signaling Technology	1/1000	-
EEA1	ab2900	Abcam	-	1/100
Erk2	K-23	Santa Cruz Biotechnology	1/1000	-
Erk[pThr202/pTyr204]	E10	Cell Signaling Technology	1/1000	-
GM130	35	BD Transduction Laboratories	-	1/250
Jak2	C-20	Santa Cruz Biotechnology	1/1000	-
LAMP1	L1418	Sigma-Aldrich	1/1000	1/250
p85	#06-195	Millipore	1/1000	1/250
pTyr	4G10	-	1/1000	-
Rab11	ab65200	Abcam	-	1/100
STAT3	C-20	Santa Cruz Biotechnology	1/1000	-
STAT5	C-17	Santa Cruz Biotechnology	1/1000	1/250
STAT5[pTyr694]	D47E7	Cell Signaling Technology	1/1000	-
TfR	ab84036	Abcam	1/1000	1/250
TGN46	ab76282	Abcam	-	1/100
Tsg101	ab30871	Abcam	1/1000	1/250
Ubiquitin	FK2	Enzo	1/1000	-

*diluted in PBS supplemented with 0.1% saponin and 10% skimmed milk

Supplementary Table 1 I List of antibodies. The list shows antibodies with source and conditions of Western blotting (WB) and immunofluorescence (IF).
